# Supplementary material for: Trends in COVID-19 hospital outcomes in England before and after vaccine introduction, a cohort study
Source: Nat Commun. 2022 Aug 17;13:4834. doi: 10.1038/s41467-022-32458-y (PMC9382625; doi:10.1038/s41467-022-32458-y)
Supplement: Supplementary file 1 — Supplementary Information [file 41467_2022_32458_MOESM1_ESM.pdf]

Supplementary Information: Trends in COVID-19 hospital outcomes in England before and after vaccine introduction, a cohort study

PD Kirwan[1], A Charlett[2], P Birrell[2], S Elgohari[2], R Hope[2], S Mandal[2], D De Angelis[1,2,*], and AM Presanis[1,*]

[1]Medical Research Council Biostatistics Unit, School of Clinical Medicine, University of Cambridge, Cambridge, UK

[2]UK Health Security Agency, London, UK

[*]These authors jointly supervised this work.

Aalen-Johansen estimator

If the competing risks of events are not independent, and the magnitude of the competing risk is large, the assumptions of conventional survival analysis methods such as Kaplan-Meier and Cox proportional hazards regression may provide biased estimates of risk, or of covariate effects on the rate of events, respectively [1]. In the case of mortality within 90 days of hospitalisation with COVID-19, the competing risk of discharge may well be large, e.g. for individuals discharged to palliative care.

The Aalen-Johansen estimator is the standard non-parametric estimate of the cumulative incidence function for competing risk [2], also described as the matrix version of the Kaplan-Meier estimator.

Let the transition hazard from state $i \in S$ to state $j \in S$, $i \neq j$ be defined as:

$$\alpha_{i,j}(t)dt = P(X_{t+dt} = j \mid X_t = i)$$

and let $\mathbf{A}(t)$ be the matrix of cumulative transition hazards:

$$A_{i,j}(t) = \int_0^t \alpha_{i,j}(u)du$$
$$A_{i,i}(t) = -\sum_{j \neq i} A_{i,j}(t)$$

then, defining as $P_{i,j}(s,t) = P(X_t = j \mid X_s = i)$, for $i, j \in S$, $s \leq t$ the probability that an individual in state s at time i will be in state t at time j, the Aalen-Johansen estimator is given by the matrix of transition probabilities:

$$\mathbf{P}(s,t) = \prod_{s,t} (\mathbf{I} + d\mathbf{A}(u))$$

The cumulative incidence function is a special case of the Aalen-Johansen estimate where:

$$C_k(t) = \int_0^t \alpha_k(u)S(u)du$$

α_k is the incidence function for outcome k, and $S(u)$ is the overall survival curve.

Fine-Gray proportional hazards regression

The Fine-Gray model estimates the hazard of a competing event (so-termed the sub-distribution hazard) among the risk set of those yet to experience an event of the type of interest by time t [3]. The risk set therefore consists of both those who have yet to experience any event and those who have yet to experience the event of interest (e.g. death) but have experienced a competing event (e.g. discharge).

The subdistribution hazard is defined as the instantaneous risk of dying (from a cause k) given that the individual has not already died:

$$h_k(t) = \lim_{\delta \to 0} \left\{ \frac{P(t \leq T < t + \delta t, K = k \mid T > t \text{ or } (T \leq t \ \& \ K \neq k))}{\delta t} \right\}$$

Fine-Gray regression links the subdistribution hazard to the Aalen-Johansen cumulative incidence estimator through the relationship:

$$h_k(t) = -\frac{d \log (1 - C_k(t))}{dt}$$

Covariate effects on the sub-distribution hazard can then be interpreted as covariate effects on the cumulative incidence, or marginal probability, of a competing event (in this case hospitalised fatality).

Stratification

Stratified survival analyses enable appropriate adjustment for important confounders, by allowing the baseline hazard to vary across strata [4]. Stratification is a similar principle to matched designs, except rather than a $1 : n$ ratio of cases to controls, as many ($a : b$ for a and b both ≥ 1) cases and controls as possible within each strata are used.

For the regression on month of admission, stratification was by age group, region of residence and vaccination status, with regression adjustment (main effects) on sex, ethnicity, index of multiple deprivation (IMD) quintile and Charlson comorbidity index (CCI). For the regression on vaccination status, stratification was by age group, region of residence and month of hospital admission, with regression adjustment (main effects) similarly on sex, ethnicity, IMD quintile and CCI.

Consistency in model estimates

Supplementary figure 7 demonstrates the high degree of agreement between the Aalen-Johansen and Fine-Gray model estimates.

Epidemic phase bias

A form of bias, often ignored in epidemic studies, is the relationship between the time from infection to symptom onset, and an individual's eventual outcome: e.g. those who go on to die may experience more rapid onset of symptoms following infection. Since estimates must be conditioned on an observed quantity (e.g. symptom onset date or hospital admission date) rather than the unobserved infection date, this relationship can introduce bias into results when an epidemic is in a mode of growth or decline [5]. The resulting bias has been termed "epidemic phase bias" and may result in hazards being over or under-estimated.

To correct for this bias, a time shift of c days should be added to records with the outcome of interest (e.g. mortality), where c is the mean difference in time from infection to symptom onset date between those experiencing the outcome and those not, as proposed by Seaman et al. [5]. As the value of c is typically unknown, sensitivity analysis with differing values of c can be used to assess the susceptibility of results to this bias.

For the sensitivity analysis in this study we shifted the date of symptom onset backwards in time by $c = 0, 1, 2, 3, 4$ days for those who died, to mitigate against the effect of more rapid symptom onset for those with more severe illness, where the shift c represents the average difference in time from infection to symptom onset between those patients who died and those who did not. The effect of this shift is shown in Supplementary Figure 8.

Supplementary figures

Supplementary figure 1: Hospitalised fatality risk (panel a) and median length of stay (panel b) by month of admission and sex.

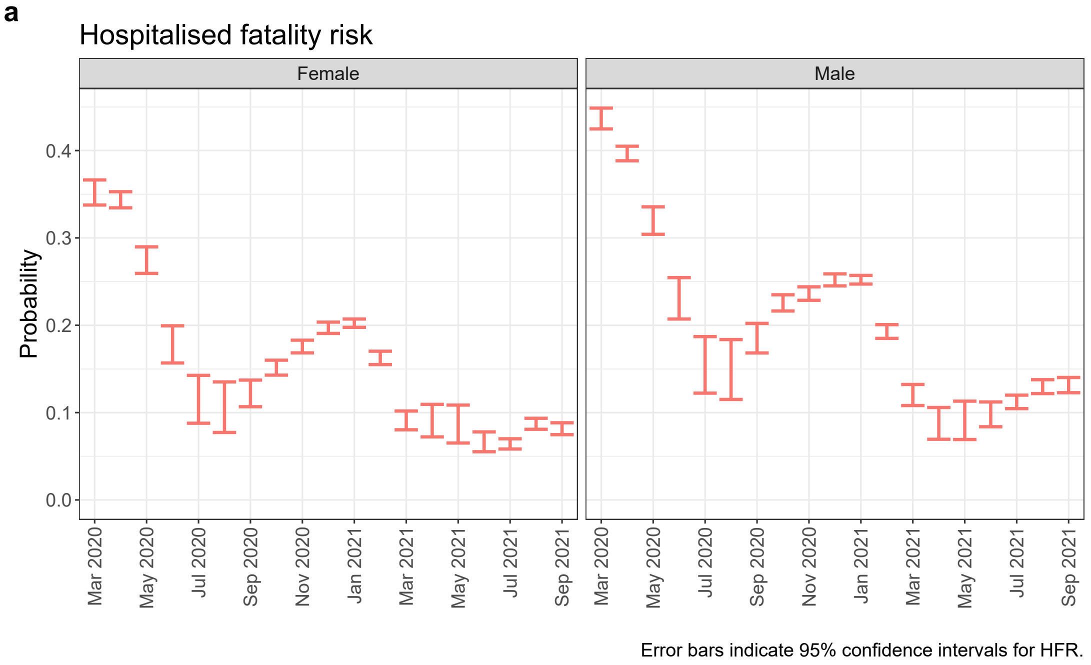

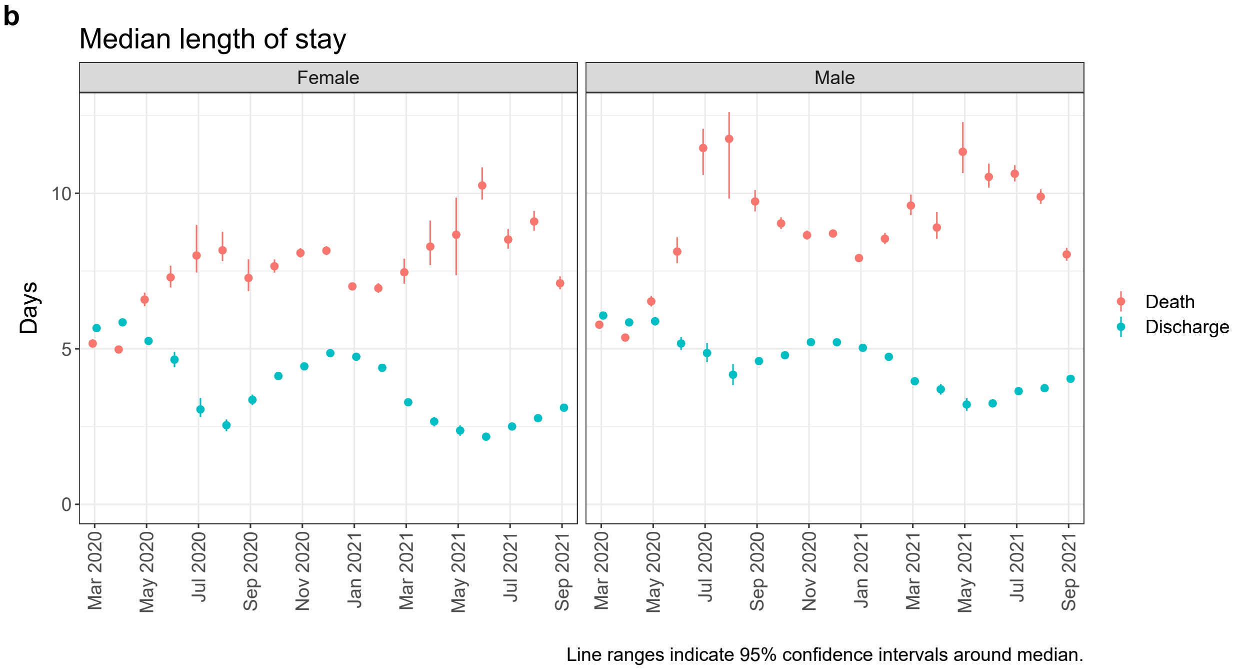

March 2020 to September 2021. Unadjusted for other covariates. $n = 259,727$ individuals with sex reported. Error bars are 95% confidence intervals.

Supplementary figure 2: Hospitalised fatality risk (panel a) and median length of stay (panel b) by month of admission and ethnicity.

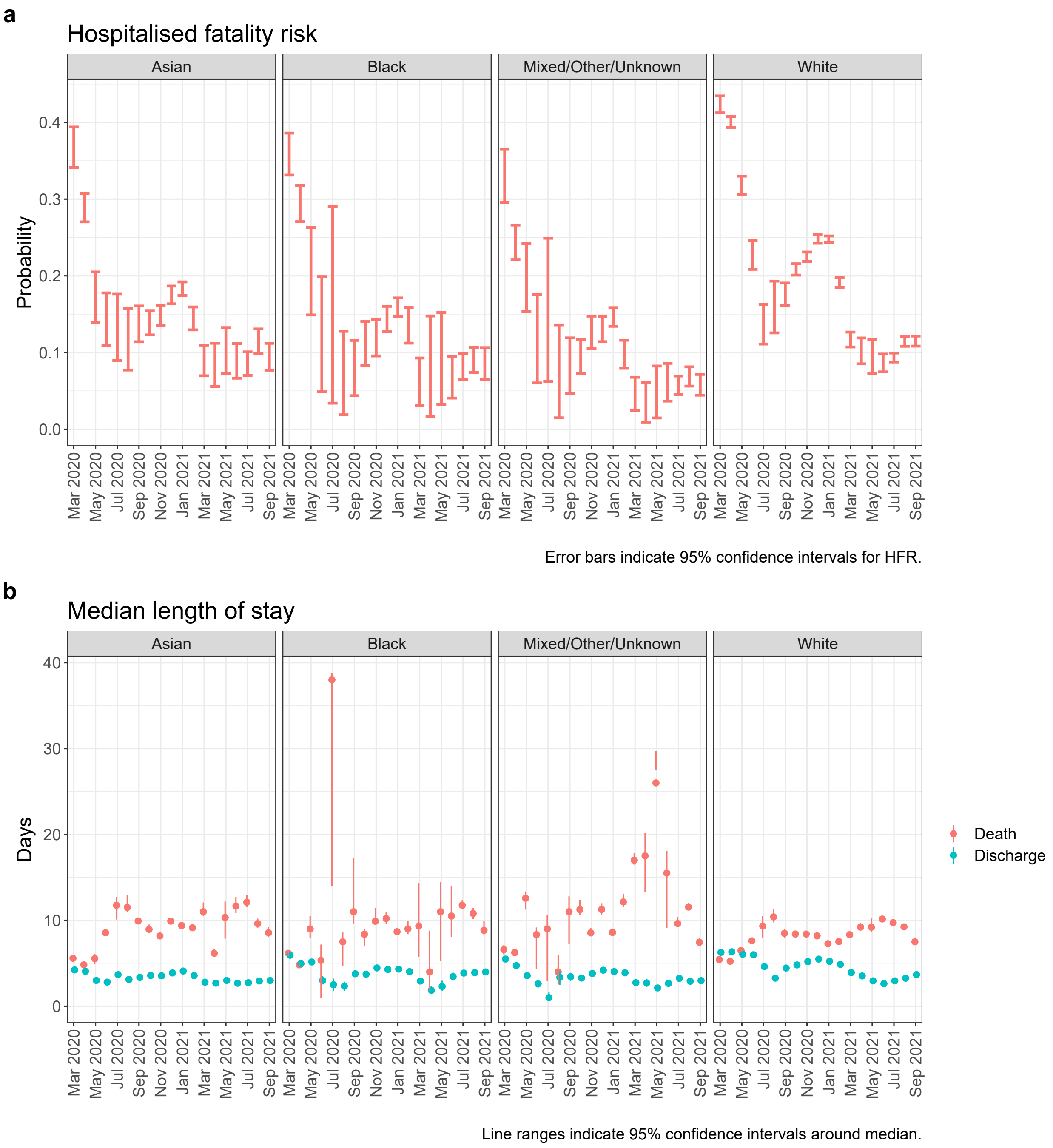

March 2020 to September 2021. Unadjusted for other covariates. $n = 259,727$ individuals with ethnicity reported. Error bars are 95% confidence intervals.

Supplementary figure 3: Hospitalised fatality risk (panel a) and median length of stay (panel b) by month of admission and region of residence.

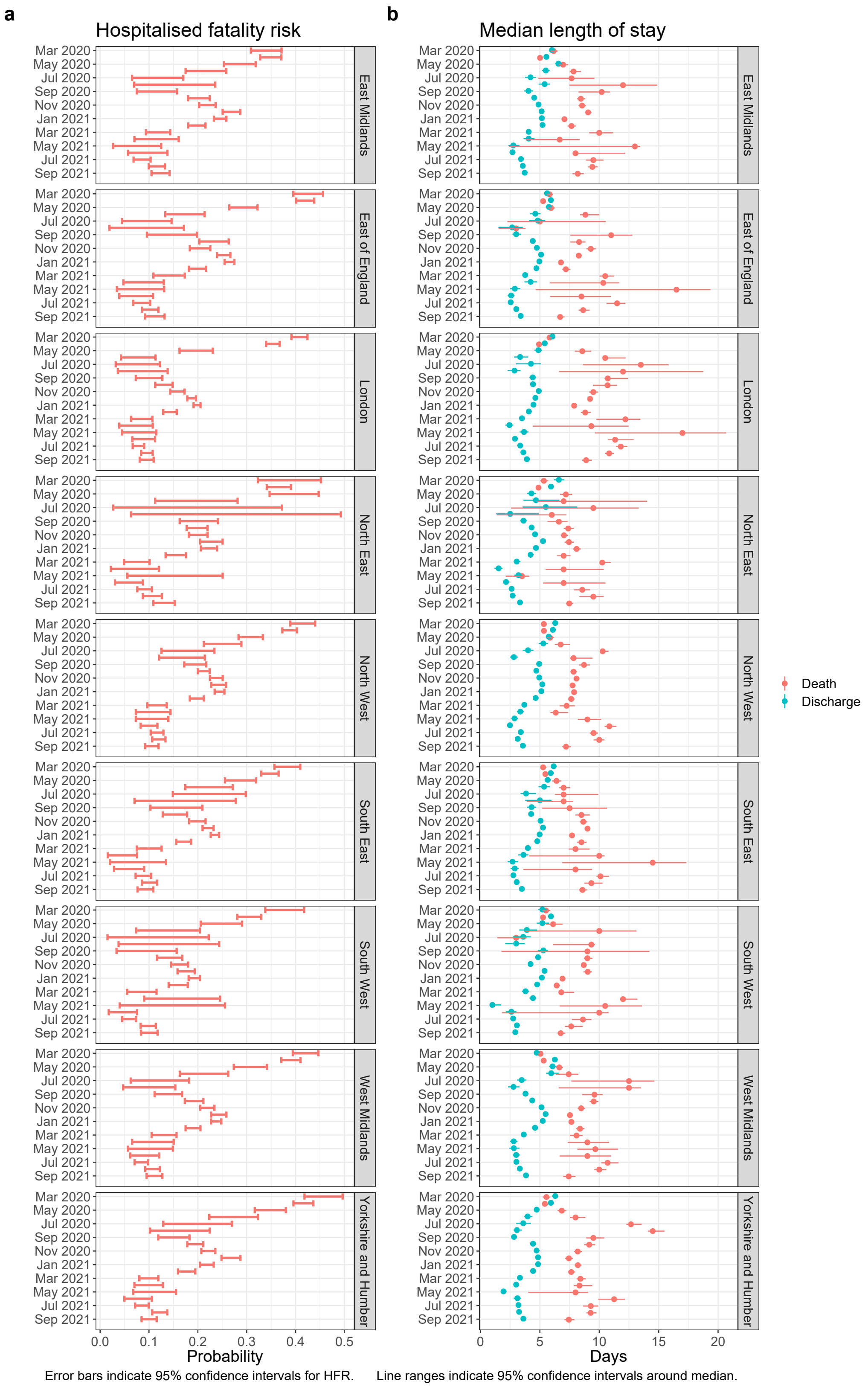

March 2020 to September 2021. Unadjusted for other covariates. $n = 259,727$ individuals with region of residence reported. Error bars are 95% confidence intervals.

Supplementary figure 4: Hospitalised fatality risk (panel a) and median length of stay (panel b) by month of admission and CCI.

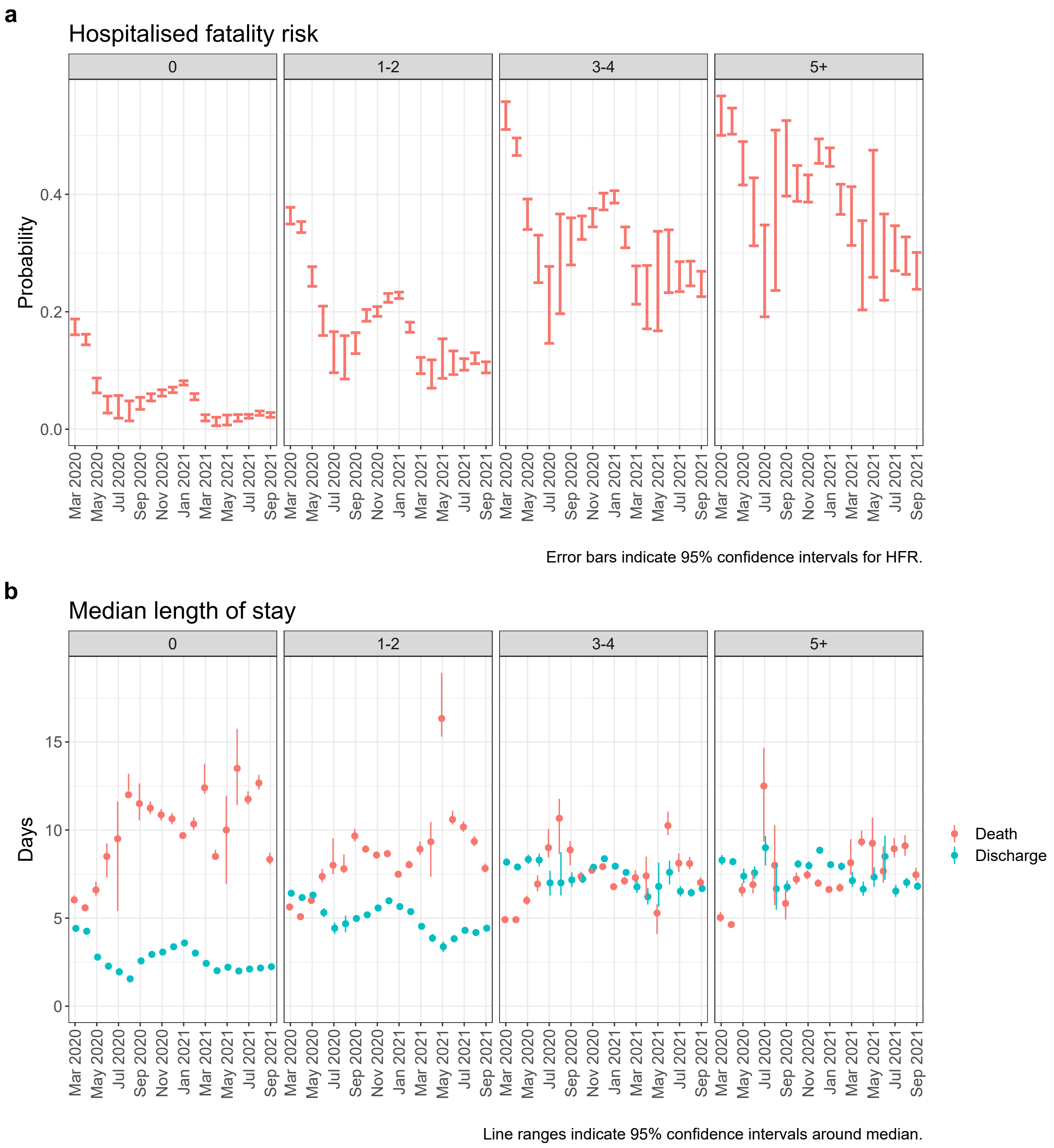

March 2020 to September 2021. Unadjusted for other covariates. $n = 238,906$ individuals with comorbidity information reported. Error bars are 95% confidence intervals.

Supplementary figure 5: Hospitalised fatality risk (panel a) and median length of stay (panel b) by month of admission and measure of hospital load.

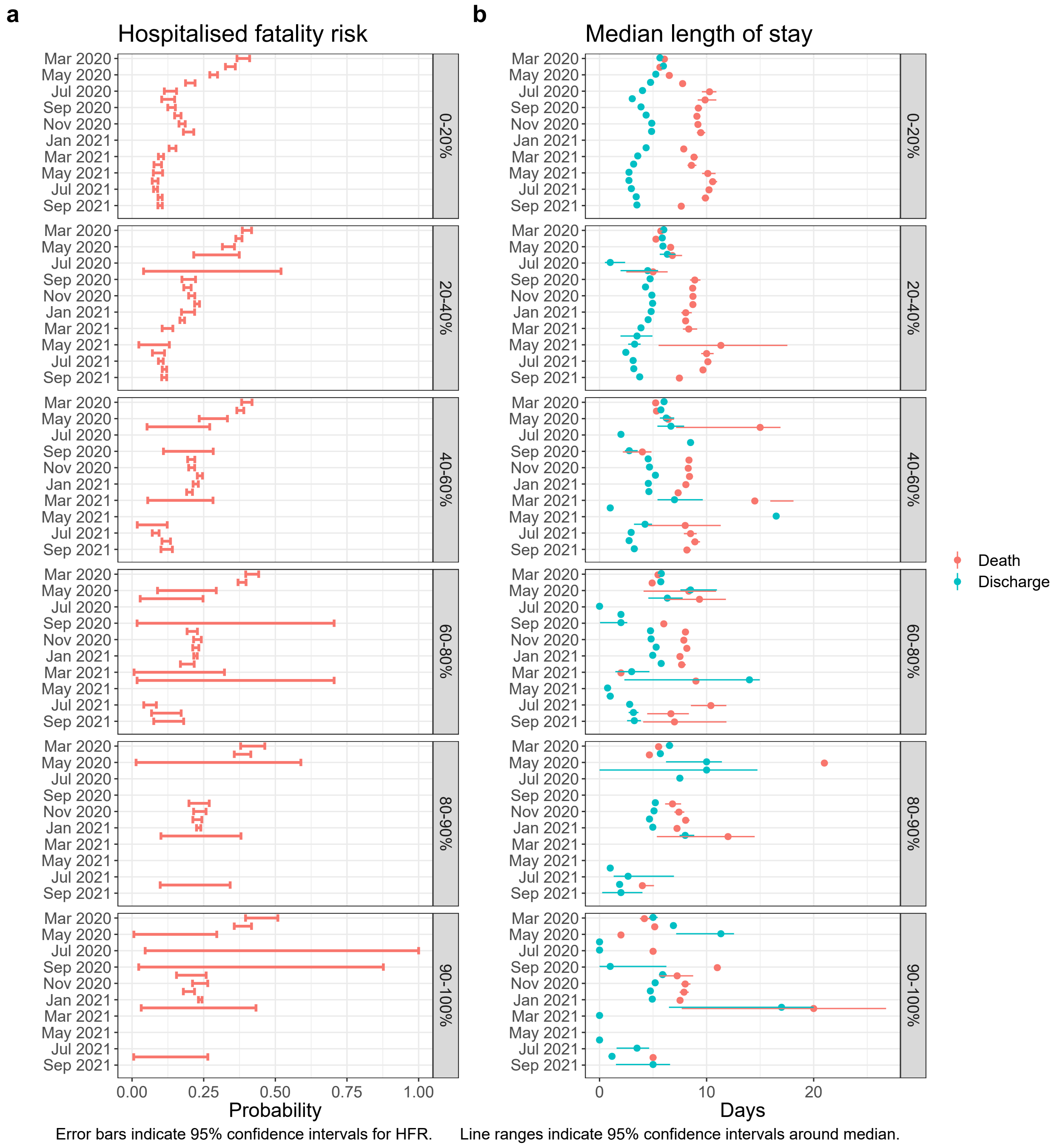

March 2020 to September 2021. Unadjusted for other covariates. $n = 259,727$ individuals with hospital admission date reported. Error bars are 95% confidence intervals.

Supplementary figure 6: Hospitalised fatality sub-distribution hazard ratios for sex, ethnicity, IMD quintile, hospital load and CCI.

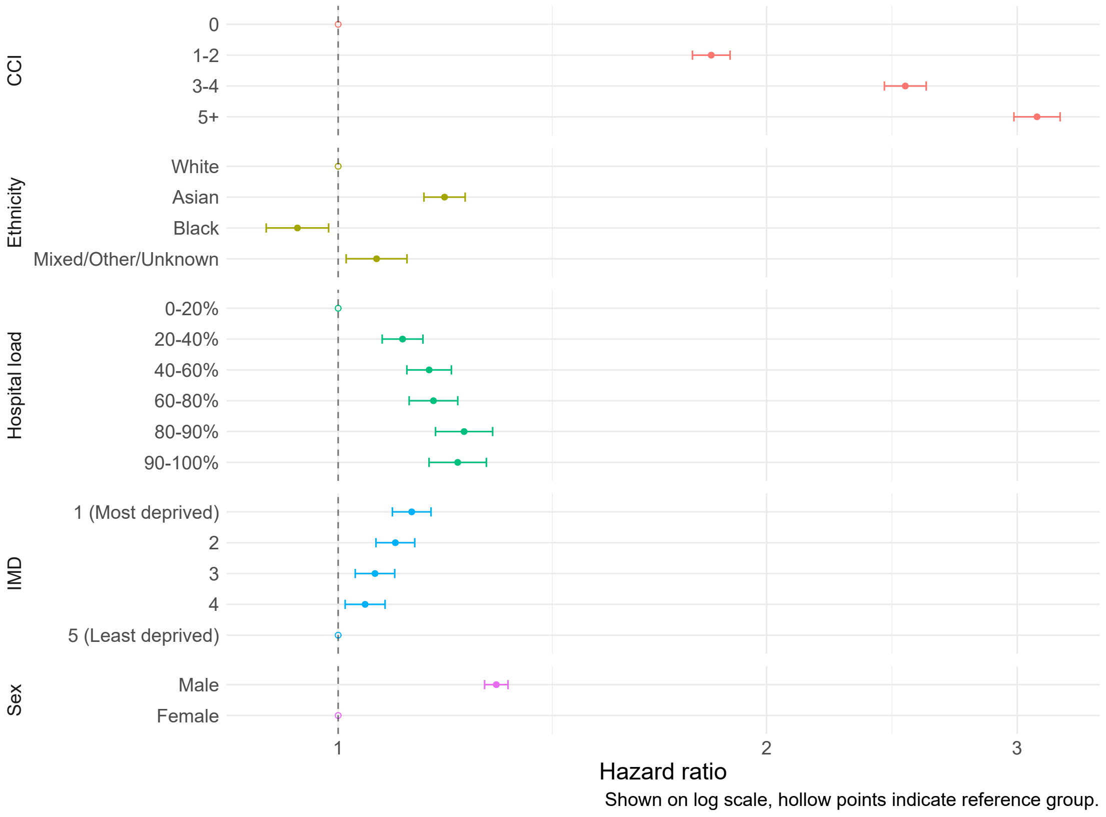

March 2020 to September 2021. Model includes stratification on age group, region of residence, and vaccination status, and regression adjustment (main effects) on month of hospital admission, sex, ethnicity, IMD quintile, hospital load, and CCI. $n = 238,897$ individuals with necessary information reported. Figure shows point estimate of hazard ratio with 95% confidence intervals.

Supplementary figure 7: Aalen-Johansen and Fine-Gray cumulative fatality estimates for first 60 days following hospital admission for selected months.

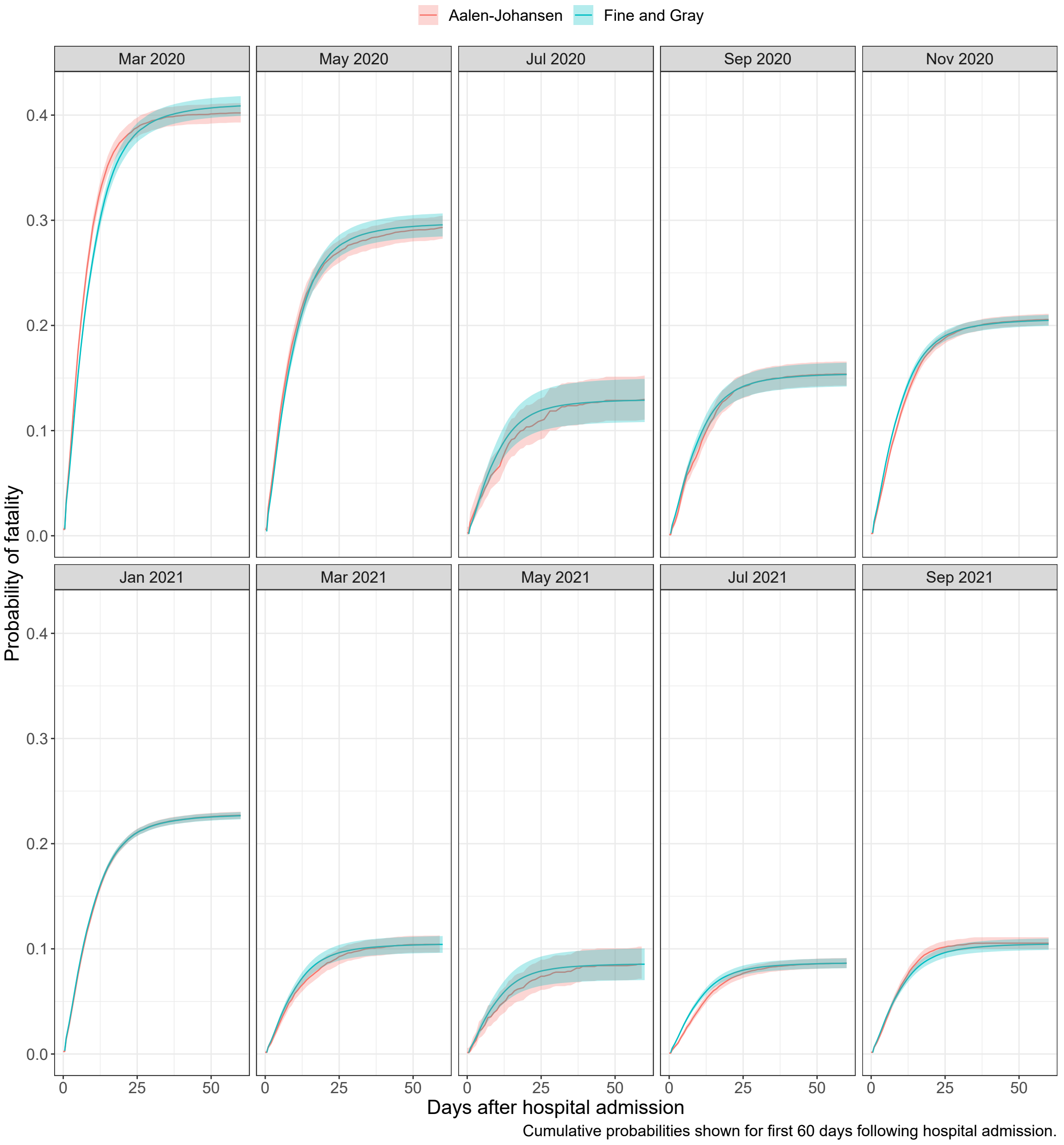

March 2020 to September 2021. $n = 259,727$ individuals. Error bands are 95% confidence intervals. Figure shows high degree of agreement between the two models.

Supplementary figure 8: Hospitalised fatality sub-distribution hazard ratio by month of symptom onset and sensitivity.

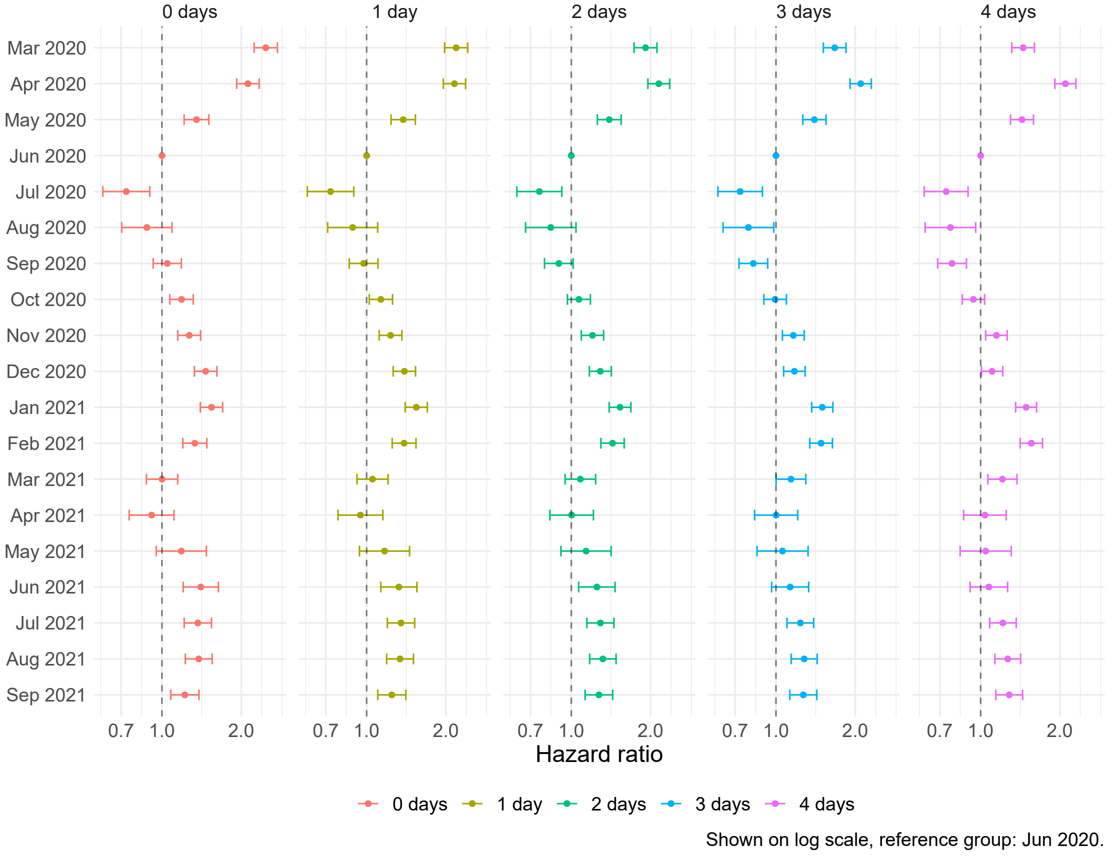

March 2020 to September 2021. Model includes stratification on age group, region of residence, and vaccination status, and regression adjustment (main effects) on month of hospital admission, sex, ethnicity, IMD quintile, hospital load, and CCI. Figure shows results of sensitivity analyses where the date of symptom onset was shifted backwards in time by $c = 0, 1, 2, 3, 4$ days for those who died, but not for those who were discharged. Reference group: June 2020. $n = 259,727$ individuals with sex reported. Figure shows point estimate of hazard ratio with 95% confidence intervals.

Supplementary tables

Supplementary table 1: Hospital outcomes according to person characteristic. Figures in brackets represent 95% confidence intervals.

Characteristic	Death	Discharge	Right-censored in hospital
**Age**			
0-14	13 (0.2%)	6272 (94.3%)	365 (5.5%)
15-24	42 (0.5%)	8509 (94.8%)	421 (4.7%)
35-44	735 (1.7%)	40,722 (92.4%)	2637 (6.0%)
45-64	7310 (9.8%)	61,162 (82.4%)	5786 (7.8%)
65-74	10,222 (24.2%)	29,369 (69.4%)	2716 (6.4%)
75-84	17,060 (35.7%)	28,133 (58.9%)	2590 (5.4%)
85+	16,566 (46.5%)	17,496 (49.1%)	1601 (4.5%)
**Sex**			
Male	30,744 (22.7%)	96,043 (70.9%)	8632 (6.4%)
Female	21,204 (17.1%)	95,620 (76.9%)	7484 (6.0%)
**Ethnicity**			
White	42,799 (21.9%)	141,406 (72.3%)	11,291 (5.8%)
Asian	5011 (15.6%)	24,910 (77.4%)	2270 (7.1%)
Black	2220 (14.4%)	11,738 (76.3%)	1436 (9.3%)
Mixed/Other/Unknown	1918 (11.5%)	13,609 (81.8%)	1119 (6.7%)
**Region of residence**			
London	8519 (18.2%)	34,288 (73.2%)	4047 (8.6%)
East Midlands	4633 (20.5%)	16,711 (74.0%)	1227 (5.4%)
East of England	6194 (23.5%)	18,971 (72.1%)	1158 (4.4%)
North East	2858 (18.9%)	11,688 (77.2%)	585 (3.9%)
North West	9016 (21.0%)	31,020 (72.1%)	2981 (6.9%)
South East	6568 (20.1%)	23,967 (73.2%)	2205 (6.7%)
South West	2874 (16.5%)	14,030 (80.7%)	487 (2.8%)
West Midlands	6177 (20.7%)	21,996 (73.8%)	1635 (5.5%)
Yorkshire and Humber	5109 (19.7%)	18,992 (73.4%)	1791 (6.9%)
**Index of multiple deprivation**			
1st quintile (most deprived)	13,715 (18.8%)	54,719 (74.9%)	4666 (6.4%)
2nd quintile	11,808 (19.8%)	44,100 (73.8%)	3846 (6.4%)
3rd quintile	9834 (20.3%)	35,572 (73.4%)	3052 (6.3%)
4th quintile	9066 (21.3%)	31,031 (72.8%)	2511 (5.9%)
5th quintile (least deprived)	7525 (21.0%)	26,241 (73.3%)	2041 (5.7%)
**Month of hospital admission**			
Mar-20	4386 (35.3%)	6494 (52.3%)	1528 (12.3%)

Supplementary table 1 continued from previous page

Apr-20	8767 (33.9%)	14,704 (56.8%)	2396 (9.3%)
May-20	1976 (26.4%)	4706 (63.0%)	793 (10.6%)
Jun-20	497 (18.4%)	1970 (73.0%)	231 (8.6%)
Jul-20	130 (12.1%)	868 (80.6%)	79 (7.3%)
Aug-20	104 (11.4%)	745 (81.8%)	62 (6.8%)
Sep-20	587 (14.9%)	3212 (81.4%)	146 (3.7%)
Oct-20	2774 (18.2%)	11,770 (77.3%)	691 (4.5%)
Nov-20	4591 (19.8%)	17,597 (75.8%)	1030 (4.4%)
Dec-20	6687 (21.3%)	23,060 (73.3%)	1721 (5.5%)
Jan-21	13,035 (21.6%)	44,028 (72.9%)	3326 (5.5%)
Feb-21	3311 (17.0%)	15,294 (78.4%)	898 (4.6%)
Mar-21	582 (10.0%)	4965 (85.5%)	262 (4.5%)
Apr-21	160 (8.3%)	1683 (87.2%)	86 (4.5%)
May-21	112 (8.2%)	1194 (87.2%)	64 (4.7%)
Jun-21	284 (7.6%)	3271 (87.1%)	201 (5.4%)
Jul-21	1149 (8.2%)	12,116 (86.6%)	719 (5.1%)
Aug-21	1556 (10.0%)	13,127 (84.3%)	895 (5.7%)
Sep-21	1260 (9.6%)	10,859 (82.8%)	988 (7.5%)
**Vaccination status at date of admission (for admissions occurring January to September 2021)**			
Unvaccinated	14,360 (14.7%)	77,720 (79.8%)	5361 (5.5%)
<21 days after first dose	2895 (26.9%)	7369 (68.4%)	510 (4.7%)
≥21 days after first dose	1346 (17.1%)	6181 (78.4%)	358 (4.5%)
≥14 days after second dose	2844 (14.7%)	15,262 (79.0%)	1208 (6.3%)
**Charleson comorbidity index**			
0	5837 (6.3%)	86,731 (93.5%)	185 (0.2%)
1-2	20,045 (21.5%)	73,062 (78.2%)	329 (0.4%)
3-4	13,216 (37.2%)	22,125 (62.3%)	186 (0.5%)
5+	7384 (43.0%)	9745 (56.7%)	61 (0.4%)
**Hospital load at time of admission (as proportion of busiest week)**			
0-20%	8815 (14.4%)	48,676 (79.3%)	3915 (6.4%)
20-40%	12,784 (19.8%)	47,937 (74.1%)	4001 (6.2%)
40-60%	11,041 (22.3%)	35,598 (71.9%)	2890 (5.8%)
60-80%	9345 (23.1%)	28,562 (70.7%)	2478 (6.1%)
80-90%	4641 (22.9%)	14,302 (70.7%)	1285 (6.4%)
90-100%	5322 (22.7%)	16,588 (70.7%)	1547 (6.6%)
**Route of admission***			
Via emergency ward	44,907 (22.0%)	143,287 (70.2%)	15,957 (7.8%)
Direct to hospital	7041 (12.7%)	48,376 (87.0%)	159 (0.3%)

*Assessed through existence of matched record in ECDS dataset.

Supplementary table 2: Hospitalised fatality risk and median length of stay (days) in hospital by month of hospital admission (unadjusted for other covariates). Figures in brackets represent 95% confidence intervals.

Month of hospital admission	Hospitalised fatality risk	Median LOS prior to death	Median LOS prior to discharge
**Mar-20**	40.3% (39.4 - 41.3%)	5.6 (5.5 - 5.6)	5.9 (5.8 - 6.0)
**Apr-20**	37.4% (36.7 - 38.0%)	5.2 (5.2 - 5.3)	5.8 (5.8 - 5.9)
**May-20**	29.7% (28.6 - 30.8%)	6.5 (6.4 - 6.7)	5.6 (5.5 - 5.7)
**Jun-20**	20.3% (18.8 - 21.9%)	7.7 (7.5 - 8.0)	4.9 (4.8 - 5.1)
**Jul-20**	13.1% (11.1 - 15.3%)	10.4 (9.7 - 11.0)	4.0 (3.8 - 4.2)
**Aug-20**	12.3% (10.3 - 14.8%)	9.9 (9.2 - 10.9)	3.1 (2.9 - 3.3)
**Sep-20**	15.5% (14.4 - 16.7%)	9.0 (8.7 - 9.3)	4.1 (4.0 - 4.2)
**Oct-20**	19.1% (18.5 - 19.7%)	8.5 (8.4 - 8.7)	4.5 (4.5 - 4.6)
**Nov-20**	20.7% (20.2, 21.3%)	8.4 (8.3 - 8.5)	4.8 (4.8 - 4.9)
**Dec-20**	22.5% (22.1 - 23.0%)	8.5 (8.4 - 8.6)	5.0 (5.0 - 5.1)
**Jan-21**	22.8% (22.5 - 23.2%)	7.5 (7.5 - 7.6)	4.9 (4.9 - 4.9)
**Feb-21**	17.8% (17.3 - 18.4%)	7.8 (7.6 - 7.9)	4.6 (4.5 - 4.6)
**Mar-21**	10.5% (9.7 - 11.4%)	8.7 (8.4 - 9.0)	3.6 (3.6 - 3.7)
**Apr-21**	8.7% (7.5 - 10.1%)	8.6 (8.3 - 9.1)	3.2 (3.1 - 3.3)
**May-21**	8.6% (7.2 - 10.3%)	10.0 (9.5 - 10.7)	2.8 (2.7 - 2.9)
**Jun-21**	8.1% (7.2 - 9.0%)	10.4 (10.1 - 10.8)	2.7 (2.6 - 2.8)
**Jul-21**	8.7% (8.3 - 9.2%)	9.9 (9.7 - 10.2)	3.0 (3.0 - 3.1)
**Aug-21**	10.7% (10.2 - 11.2%)	9.6 (9.4 - 9.8)	3.2 (3.2 - 3.3)
**Sep-21**	10.5% (10.0 - 11.1%)	7.6 (7.5 - 7.8)	3.6 (3.5 - 3.6)

Supplementary table 3: Hospitalised fatality risk and median length of stay (days) in hospital prior to death or discharge by month of hospital admission and age group (unadjusted for other covariates). Estimates are replaced with a dash (-) where insufficient information was available. Figures in brackets represent 95% confidence intervals.

**Month of hospital admission**	**0-14**	**15-24**	**25-44**	**45-64**
**Mar-20**				
HFR	1.4% (0.4 - 5.7%)	4.0% (1.7 - 9.4%)	5.7% (4.5 - 7.3%)	22.3% (20.9 - 23.9%)
Median LOS (death)	6.0 (2.9 - 7.8)	4.0 (1.4 - 5.9)	7.7 (7.1 - 8.3)	7.5 (7.3 - 7.9)
Median LOS (discharge)	1.0 (0.9 - 1.1)	2.4 (2.0 - 2.8)	3.0 (2.8 - 3.2)	5.6 (5.4 - 5.7)
**Apr-20**				
HFR	0.5% (0.1 - 3.9%)	2.6% (1.1 - 6.2%)	5.0% (4.1 - 6.1%)	18.2% (17.3 - 19.2%)
Median LOS (death)	5.0 (5.0 - 5.0)	4.7 (0.8 - 8.9)	5.3 (5.1 - 5.6)	6.7 (6.5 - 6.8)
Median LOS (discharge)	1.4 (1.1 - 1.7)	1.6 (1.3 - 1.9)	2.9 (2.8 - 3.1)	4.8 (4.7 - 4.8)
**May-20**				
HFR	1.0% (0.1 - 6.7%)	0.7% (0.1 - 5.3%)	1.9% (1.1 - 3.2%)	13.3% (11.6 - 15.3%)
Median LOS (death)	6.0 (6.0 - 6.0)	3.0 (3.0 - 3.0)	8.5 (8.3 - 10.2)	7.8 (7.4 - 8.4)
Median LOS (discharge)	1.1 (0.9 - 1.4)	1.0 (0.9 - 1.3)	1.8 (1.6 - 1.9)	4.3 (4.2 - 4.5)
**Jun-20**				
HFR	1.7% (0.2 - 11.6%)	1.4% (0.2 - 9.6%)	1.2% (0.5 - 3.3%)	7.9% (5.9 - 10.5%)
Median LOS (death)	2.0 (2.0 - 2.0)	16.0 (16.0 - 16.0)	0.8 (0.6 - 1.6)	8.4 (8.0 - 9.3)
Median LOS (discharge)	0.9 (0.8 - 1.2)	1.6 (1.1 - 2.0)	1.4 (1.2 - 1.6)	3.9 (3.5 - 4.2)
**Jul-20**				
HFR	-	-	-	8.8% (5.9 - 13.0%)
Median LOS (death)	-	-	-	14.0 (11.9 - 16.3)
Median LOS (discharge)	-	-	-	4.7 (4.3 - 5.0)
**Aug-20**				
HFR	-	-	1.0% (0.3 - 4.0%)	7.9% (5.1 - 12.3%)
Median LOS (death)	-	-	12.0 (8.1 - 14.2)	12.0 (10.1 - 13.9)
Median LOS (discharge)	-	-	1.4 (1.1 - 1.7)	3.9 (3.4 - 4.3)
**Sep-20**				
HFR	-	0.5% (0.1 - 3.8%)	0.7% (0.3 - 1.6%)	4.8% (3.7 - 6.3%)
Median LOS (death)	-	18.0 (18.0 - 18.0)	11.0 (7.6 - 14.1)	13.6 (12.5 - 14.4)
Median LOS (discharge)	-	1.0 (0.9 - 1.1)	2.1 (1.9 - 2.2)	4.7 (4.5 - 4.9)
**Oct-20**				
HFR	-	0.2% (0.0 - 1.3%)	1.2% (0.8 - 1.8%)	8.1% (7.3 - 9.0%)
Median LOS (death)	-	10.0 (10.0 - 10.0)	7.5 (5.6 - 8.3)	12.6 (12.2 - 13.0)
Median LOS (discharge)	-	0.9 (0.9 - 1.0)	2.0 (1.9 - 2.2)	4.3 (4.2 - 4.4)
**Nov-20**				
HFR	0.2% (0.0 - 1.6%)	0.5% (0.2 - 1.5%)	1.4% (1.1 - 1.9%)	8.6% (8.0 - 9.4%)
Median LOS (death)	0.5 (0.5 - 0.5)	5.0 (3.5 - 5.8)	10.0 (9.4 - 11.0)	12.7 (12.3 - 13.1)
Median LOS (discharge)	0.9 (0.9 - 0.9)	1.0 (0.9 - 1.1)	2.1 (2.0 - 2.2)	4.5 (4.4 - 4.5)
**Dec-20**				
HFR	-	0.3% (0.1 - 1.1%)	2.0% (1.6 - 2.4%)	10.1% (9.5 - 10.7%)
Median LOS (death)	-	9.5 (7.6 - 10.6)	13.6 (13.0 - 14.4)	12.4 (12.1 - 12.7)
Median LOS (discharge)	-	1.2 (1.0 - 1.3)	2.4 (2.3 - 2.5)	4.8 (4.7 - 4.8)
**Jan-21**				
HFR	0.2% (0.1 - 1.0%)	0.7% (0.4 - 1.3%)	2.2% (1.9 - 2.5%)	10.8% (10.3 - 11.3%)
Median LOS (death)	16.0 (16.0 - 16.0)	8.0 (3.8 - 9.8)	12.4 (11.8 - 13.0)	11.6 (11.4 - 11.8)

Supplementary table 3 continued from previous page

Median LOS (discharge)	0.9 (0.9 - 1.0)	1.3 (1.2 - 1.4)	2.8 (2.7 - 2.8)	4.6 (4.6 - 4.7)
**Feb-21**				
HFR	-	-	1.7% (1.3 - 2.2%)	9.5% (8.8 - 10.3%)
Median LOS (death)	-	-	7.0 (6.5 - 9.8)	11.6 (11.2 - 11.9)
Median LOS (discharge)	-	-	2.3 (2.3 - 2.4)	4.4 (4.3 - 4.5)
**Mar-21**				
HFR	-	-	1.2% (0.7 - 1.9%)	5.8% (4.8 - 6.9%)
Median LOS (death)	-	-	7.0 (3.5 - 12.8)	13.6 (12.9 - 15.2)
Median LOS (discharge)	-	-	2.0 (1.9 - 2.2)	4.0 (3.8 - 4.1)
**Apr-21**				
HFR	-	-	0.3% (0.1 - 1.4%)	4.6% (3.1 - 6.8%)
Median LOS (death)	-	-	19.5 (9.8 - 25.0)	13.0 (12.5 - 14.3)
Median LOS (discharge)	-	-	2.0 (1.8 - 2.3)	3.6 (3.4 - 3.8)
**May-21**				
HFR	-	-	1.0% (0.4 - 2.4%)	9.6% (6.8 - 13.5%)
Median LOS (death)	-	-	14.0 (7.9 - 20.8)	18.0 (15.0 - 18.8)
Median LOS (discharge)	-	-	2.4 (2.2 - 2.7)	4.0 (3.7 - 4.3)
**Jun-21**				
HFR	0.9% (0.2 - 3.5%)	-	1.2% (0.8 - 2.0%)	9.7% (7.9 - 12.0%)
Median LOS (death)	7.5 (4.8 - 9.0)	-	19.0 (17.5 - 20.5)	13.6 (11.6 - 15.2)
Median LOS (discharge)	0.9 (0.8 - 0.9)	-	2.2 (2.1 - 2.4)	4.0 (3.8 - 4.2)
**Jul-21**				
HFR	-	0.3% (0.1 - 0.8%)	1.5% (1.2 - 1.9%)	8.0% (7.1 - 9.0%)
Median LOS (death)	-	22.5 (17.8 - 25.9)	15.5 (13.9 - 16.7)	13.1 (12.7 - 13.6)
Median LOS (discharge)	-	1.2 (1.1 - 1.3)	2.2 (2.1 - 2.3)	4.7 (4.6 - 4.8)
**Aug-21**				
HFR	0.2% (0.1 - 0.9%)	0.6% (0.3 - 1.3%)	1.1% (0.8 - 1.5%)	8.4% (7.6 - 9.4%)
Median LOS (death)	16.0 (8.2 - 20.4)	16.0 (7.9 - 17.6)	13.4 (12.7 - 14.0)	12.4 (11.9 - 13.2)
Median LOS (discharge)	0.9 (0.9 - 1.0)	1.2 (1.1 - 1.3)	2.0 (2.0 - 2.1)	4.2 (4.1 - 4.3)
**Sep-21**				
HFR	0.1% (0.0 - 1.0%)	0.3% (0.1 - 1.2%)	0.8% (0.5 - 1.2%)	6.7% (5.9 - 7.7%)
Median LOS (death)	9.0 (9.0 - 9.0)	19.5 (16.8 - 21.0)	11.0 (10.0 - 14.0)	10.4 (10 - 10.9)
Median LOS (discharge)	0.8 (0.8 - 0.9)	1.0 (1.0 - 1.1)	2.1 (2.0 - 2.2)	4.2 (4.0 - 4.3)

Supplementary table 3 continued

**Month of hospital admission**	**65-74**	**75-84**	**85+**
**Mar-20**			
HFR	43.2% (41.1 - 45.4%)	57.3% (55.5 - 59.2%)	66.5% (64.4 - 68.7%)
Median LOS (death)	6.2 (6.0 - 6.4)	4.9 (4.8 - 5.0)	5.0 (4.8 - 5.1)
Median LOS (discharge)	6.8 (6.6 - 7.0)	7.8 (7.6 - 8.0)	9.5 (9.3 - 9.8)
**Apr-20**			
HFR	38.2% (36.8 - 39.7%)	50.4% (49.2 - 51.7%)	60.0% (58.7 - 61.4%)
Median LOS (death)	5.6 (5.5 - 5.8)	4.8 (4.7 - 4.9)	4.9 (4.8 - 5.0)
Median LOS (discharge)	6.6 (6.5 - 6.7)	7.8 (7.7 - 7.9)	8.5 (8.4 - 8.7)
**May-20**			
HFR	27.0% (24.4 - 29.8%)	41.1% (38.8 - 43.5%)	48.5% (46.2 - 50.9%)
Median LOS (death)	6.9 (6.6 - 7.3)	5.9 (5.7 - 6.1)	6.8 (6.5 - 7.0)

Supplementary table 3 continued from previous page

Median LOS (discharge)	6.6 (6.3 - 6.9)	8.2 (7.9 - 8.4)	8.6 (8.3 - 8.8)
**Jun-20**			
HFR	20.6% (17.0 - 25.0%)	36.0% (32.3 - 40.2%)	33.6% (29.6 - 38.1%)
Median LOS (death)	9.4 (8.9 - 10.2)	7.5 (7.1 - 7.9)	7.1 (6.6 - 7.7)
Median LOS (discharge)	5.9 (5.5 - 6.3)	7.4 (7.1 - 7.7)	8.9 (8.5 - 9.3)
**Jul-20**			
HFR	15.2% (10.5 - 22.2%)	24.6% (19.2 - 31.5%)	27.4% (20.8 - 36.1%)
Median LOS (death)	11.8 (10.4 - 12.6)	7.8 (7.3 - 9.2)	6.3 (5.9 - 7.7)
Median LOS (discharge)	4.3 (3.8 - 4.8)	6.7 (6.1 - 7.3)	8.6 (7.8 - 9.5)
**Aug-20**			
HFR	19.5% (13.5 - 28.1%)	25.8% (19.3 - 34.7%)	34.2% (25.4 - 46.2%)
Median LOS (death)	11.7 (9.5 - 15.7)	12.0 (9.7 - 12.5)	8.1 (7.9 - 8.5)
Median LOS (discharge)	4.4 (3.9 - 5.3)	7.0 (6.3 - 8.3)	7.4 (6.7 - 8.4)
**Sep-20**			
HFR	23.3% (20.2 - 26.7%)	30.3% (27.0 - 33.9%)	44.7% (40.0 - 50.0%)
Median LOS (death)	12.4 (11.9 - 13.2)	7.9 (7.4 - 8.5)	6.8 (6.3 - 7.5)
Median LOS (discharge)	5.8 (5.5 - 6.1)	6.6 (6.3 - 6.9)	8.1 (7.5 - 8.8)
**Oct-20**			
HFR	21.3% (19.8 - 22.9%)	34.0% (32.4 - 35.7%)	44.7% (42.5 - 47.1%)
Median LOS (death)	10.0 (9.7 - 10.3)	8.0 (7.7 - 8.2)	7.4 (7.1 - 7.6)
Median LOS (discharge)	5.5 (5.4 - 5.6)	6.8 (6.7 - 7.0)	10.4 (10.1 - 10.7)
**Nov-20**			
HFR	23.6% (22.3 - 24.9%)	33.2% (31.9 - 34.6%)	46.0% (44.3 - 47.7%)
Median LOS (death)	9.3 (9.0 - 9.7)	7.6 (7.5 - 7.8)	7.6 (7.5 - 7.8)
Median LOS (discharge)	5.8 (5.7 - 5.9)	7.7 (7.6 - 7.8)	9.3 (9.1 - 9.6)
**Dec-20**			
HFR	26.3% (25.1 - 27.6%)	38.5% (37.3 - 39.8%)	49.4% (48.0 - 50.9%)
Median LOS (death)	9.7 (9.4 - 9.9)	7.6 (7.4 - 7.7)	7.6 (7.4 - 7.7)
Median LOS (discharge)	6.4 (6.3 - 6.5)	7.9 (7.8 - 8.0)	10.1 (10.0 - 10.3)
**Jan-21**			
HFR	26.1% (25.3 - 27.0%)	38.6% (37.7 - 39.5%)	49.5% (48.4 - 50.6%)
Median LOS (death)	8.9 (8.7 - 9.1)	6.6 (6.5 - 6.7)	6.6 (6.5 - 6.7)
Median LOS (discharge)	5.9 (5.8 - 6.0)	7.4 (7.4 - 7.5)	8.6 (8.4 - 8.7)
**Feb-21**			
HFR	21.9% (20.5 - 23.3%)	31.8% (30.2 - 33.5%)	42.9% (40.9 - 44.9%)
Median LOS (death)	8.4 (8.1 - 8.7)	7.0 (6.8 - 7.3)	6.8 (6.6 - 7.0)
Median LOS (discharge)	6.0 (5.9 - 6.1)	7.0 (6.8 - 7.1)	8.8 (8.6 - 9.0)
**Mar-21**			
HFR	17.3% (14.7 - 20.4%)	24.1% (21.0 - 27.7%)	34.9% (31.1 - 39.2%)
Median LOS (death)	10.3 (9.6 - 11.3)	7.2 (6.9 - 7.7)	7.6 (7.1 - 8.1)
Median LOS (discharge)	5.0 (4.8 - 5.2)	6.2 (5.9 - 6.6)	9.0 (8.5 - 9.4)
**Apr-21**			
HFR	15.2% (11.0 - 21.1%)	23.2% (18.1 - 29.8%)	34.9% (28.2 - 43.2%)
Median LOS (death)	9.8 (8.8 - 10.8)	7.8 (7.1 - 8.6)	6.3 (5.1 - 8.2)
Median LOS (discharge)	5.6 (5.2 - 6.1)	6.2 (5.8 - 6.7)	7.8 (7.1 - 8.5)
**May-21**			
HFR	24.2% (17.6 - 33.2%)	24.0% (17.4 - 33.2%)	27.8% (19.1 - 40.3%)
Median LOS (death)	11.0 (8.0 - 12.7)	5.9 (5.0 - 6.5)	9.6 (9.4 - 11.0)
Median LOS (discharge)	3.6 (3.2 - 4.1)	7.3 (6.5 - 8.2)	5.1 (4.4 - 5.8)
**Jun-21**			

Supplementary table 3 continued from previous page

HFR	17.6% (13.7 - 22.7%)	26.2% (21.4 - 32.2%)	43.5% (36.4 - 51.9%)
Median LOS (death)	11.4 (11.1 - 12.1)	9.0 (8.4 - 9.6)	7.9 (7.4 - 8.7)
Median LOS (discharge)	4.9 (4.5 - 5.3)	7.8 (7.0 - 8.6)	9.5 (8.8 - 10.2)
**Jul-21**			
HFR	18.4% (16.4 - 20.6%)	25.7% (23.4 - 28.2%)	35.4% (32.1 - 38.9%)
Median LOS (death)	9.7 (9.2 - 10.3)	8.0 (7.7 - 8.4)	8.6 (8.1 - 9.2)
Median LOS (discharge)	5.0 (4.8 - 5.2)	5.8 (5.6 - 6.1)	7.3 (6.8 - 7.8)
**Aug-21**			
HFR	17.7% (16.0 - 19.5%)	24.8% (22.9 - 26.7%)	34.2% (31.6 - 37.1%)
Median LOS (death)	10.6 (10.2 - 11.0)	7.6 (7.3 - 7.9)	8.5 (8.1 - 8.8)
Median LOS (discharge)	4.8 (4.7 - 5.0)	6.0 (5.8 - 6.2)	7.4 (7.0 - 7.7)
**Sep-21**			
HFR	15.2% (13.6 - 17.0%)	21.8% (20.0 - 23.7%)	31.1% (28.6 - 33.9%)
Median LOS (death)	7.6 (7.2 - 8.0)	7.4 (7.1 - 7.6)	6.8 (6.6 - 7.1)
Median LOS (discharge)	5.3 (5.1 - 5.4)	5.5 (5.3 - 5.7)	8.0 (7.6 - 8.4)

Supplementary table 4: Hospitalised fatality risk and median length of stay (days) in hospital prior to death or discharge by month of hospital admission and sex (unadjusted for other covariates). Figures in brackets represent 95% confidence intervals.

Month of hospital admission	Male	Female
**Mar-20**		
HFR	43.7% (42.5 - 44.9%)	35.2% (33.8 - 36.6%)
Median LOS (death)	5.8 (5.7 - 5.9)	5.2 (5.0 - 5.3)
Median LOS (discharge)	6.1 (6.0 - 6.2)	5.7 (5.5 - 5.8)
**Apr-20**		
HFR	39.7% (38.8 - 40.5%)	34.4% (33.4 - 35.3%)
Median LOS (death)	5.4 (5.3 - 5.4)	5.0 (4.9 - 5.1)
Median LOS (discharge)	5.8 (5.8 - 5.9)	5.8 (5.8 - 5.9)
**May-20**		
HFR	31.9% (30.4 - 33.6%)	27.4% (25.9 - 29.0%)
Median LOS (death)	6.5 (6.4 - 6.7)	6.6 (6.4 - 6.8)
Median LOS (discharge)	5.9 (5.7 - 6.0)	5.3 (5.1 - 5.4)
**Jun-20**		
HFR	23.0% (20.7 - 25.5%)	17.7% (15.7 - 19.9%)
Median LOS (death)	8.1 (7.8 - 8.6)	7.3 (7.0 - 7.7)
Median LOS (discharge)	5.2 (5.0 - 5.4)	4.7 (4.4 - 4.9)
**Jul-20**		
HFR	15.1% (12.2 - 18.7%)	11.2% (8.8 - 14.3%)
Median LOS (death)	11.5 (10.6 - 12.1)	8.0 (7.5 - 9.0)
Median LOS (discharge)	4.9 (4.6 - 5.2)	3.1 (2.8 - 3.4)
**Aug-20**		
HFR	14.5% (11.5 - 18.4%)	10.2% (7.7 - 13.5%)
Median LOS (death)	11.7 (9.8 - 12.6)	8.2 (7.8 - 8.8)
Median LOS (discharge)	4.2 (3.8 - 4.5)	2.5 (2.3 - 2.7)
**Sep-20**		
HFR	18.4% (16.8 - 20.2%)	12.1% (10.7 - 13.7%)
Median LOS (death)	9.7 (9.4 - 10.1)	7.3 (6.9 - 7.9)
Median LOS (discharge)	4.6 (4.5 - 4.7)	3.4 (3.2 - 3.5)
**Oct-20**		
HFR	22.5% (21.6 - 23.5%)	15.1% (14.3 - 16.0%)
Median LOS (death)	9.0 (8.8 - 9.2)	7.7 (7.4 - 7.9)
Median LOS (discharge)	4.8 (4.7 - 4.9)	4.1 (4.0 - 4.2)
**Nov-20**		
HFR	23.6% (22.9 - 24.4%)	17.5% (16.8 - 18.3%)
Median LOS (death)	8.7 (8.5 - 8.8)	8.1 (7.9 - 8.2)
Median LOS (discharge)	5.2 (5.1 - 5.3)	4.4 (4.4 - 4.5)
**Dec-20**		
HFR	25.2% (24.5 - 25.9%)	19.7% (19.1 - 20.4%)
Median LOS (death)	8.7 (8.6 - 8.8)	8.2 (8.0 - 8.3)
Median LOS (discharge)	5.2 (5.2 - 5.3)	4.9 (4.8 - 4.9)
**Jan-21**		

Supplementary table 4 continued from previous page

HFR	25.2% (24.7 - 25.7%)	20.2% (19.8 - 20.7%)
Median LOS (death)	7.9 (7.8 - 8.0)	7.0 (6.9 - 7.1)
Median LOS (discharge)	5.0 (5.0 - 5.1)	4.7 (4.7 - 4.8)
**Feb-21**		
HFR	19.3% (18.5 - 20.1%)	16.2% (15.5 - 17.0%)
Median LOS (death)	8.5 (8.4 - 8.7)	6.9 (6.8 - 7.1)
Median LOS (discharge)	4.7 (4.7 - 4.8)	4.4 (4.3 - 4.5)
**Mar-21**		
HFR	12.0% (10.8 - 13.2%)	9.0% (8.0 - 10.2%)
Median LOS (death)	9.6 (9.3 - 10.0)	7.5 (7.1 - 7.9)
Median LOS (discharge)	4.0 (3.9 - 4.1)	3.3 (3.2 - 3.4)
**Apr-21**		
HFR	8.6% (6.9 - 10.6%)	8.9% (7.2 - 10.9%)
Median LOS (death)	8.9 (8.5 - 9.4)	8.3 (7.7 - 9.1)
Median LOS (discharge)	3.7 (3.5 - 3.9)	2.7 (2.5 - 2.8)
**May-21**		
HFR	8.8% (6.9 - 11.3%)	8.4% (6.5 - 10.9%)
Median LOS (death)	11.3 (10.6 - 12.3)	8.7 (7.4 - 9.9)
Median LOS (discharge)	3.2 (3.0 - 3.4)	2.4 (2.2 - 2.5)
**Jun-21**		
HFR	9.7% (8.4 - 11.2%)	6.6% (5.5 - 7.8%)
Median LOS (death)	10.5 (10.2 - 11.0)	10.3 (9.8 - 10.8)
Median LOS (discharge)	3.2 (3.1 - 3.4)	2.2 (2.1 - 2.3)
**Jul-21**		
HFR	11.2% (10.5 - 12.0%)	6.4% (5.8 - 7.0%)
Median LOS (death)	10.6 (10.4 - 10.9)	8.5 (8.2 - 8.8)
Median LOS (discharge)	3.6 (3.6 - 3.7)	2.5 (2.4 - 2.6)
**Aug-21**		
HFR	12.9% (12.2 - 13.8%)	8.7% (8.1 - 9.3%)
Median LOS (death)	9.9 (9.7 - 10.1)	9.1 (8.8 - 9.4)
Median LOS (discharge)	3.7 (3.7 - 3.8)	2.8 (2.7 - 2.8)
**Sep-21**		
HFR	13.1% (12.3 - 14.0%)	8.1% (7.5 - 8.8%)
Median LOS (death)	8.0 (7.8 - 8.2)	7.1 (6.9 - 7.3)
Median LOS (discharge)	4.0 (4.0 - 4.1)	3.1 (3.0 - 3.2)

Supplementary table 5: Hospitalised fatality risk and median length of stay (days) in hospital prior to death or discharge by month of hospital admission and ethnicity (unadjusted for other covariates). Figures in brackets represent 95% confidence intervals.

Month of hospital admission	Asian	Black	White
**Mar-20**			
HFR	36.7% (34.1 - 39.4%)	35.8% (33.1 - 38.6%)	42.3% (41.2 - 43.4%)
Median LOS (death)	5.6 (5.3 - 5.9)	6.2 (5.9 - 6.5)	5.4 (5.3 - 5.5)
Median LOS (discharge)	4.2 (4.1 - 4.4)	5.9 (5.8 - 6.1)	6.3 (6.2 - 6.4)
**Apr-20**			
HFR	28.8% (27.0 - 30.7%)	29.3% (27.1 - 31.8%)	40.1% (39.3 - 40.8%)
Median LOS (death)	4.8 (4.6 - 5.0)	4.8 (4.6 - 5.0)	5.2 (5.2 - 5.3)
Median LOS (discharge)	4.1 (3.9 - 4.2)	5.0 (4.8 - 5.1)	6.3 (6.3 - 6.4)
**May-20**			
HFR	16.9% (13.9 - 20.5%)	19.8% (14.9 - 26.3%)	31.8% (30.6 - 33.0%)
Median LOS (death)	5.5 (4.9 - 6.1)	9.0 (7.9 - 10.5)	6.5 (6.3 - 6.6)
Median LOS (discharge)	3.0 (2.8 - 3.2)	5.1 (4.8 - 5.5)	6.1 (5.9 - 6.2)
**Jun-20**			
HFR	13.9% (10.9 - 17.8%)	9.9% (4.9 - 19.9%)	22.7% (20.8 - 24.6%)
Median LOS (death)	8.6 (8.3 - 8.9)	5.3 (1.0 - 7.2)	7.6 (7.3 - 7.9)
Median LOS (discharge)	2.8 (2.6 - 3.0)	3.0 (2.5 - 3.5)	6.0 (5.8 - 6.2)
**Jul-20**			
HFR	12.6% (8.9 - 17.7%)	9.9% (3.4 - 29.0%)	13.4% (11.1 - 16.3%)
Median LOS (death)	11.8 (10.1 - 12.7)	38.0 (14.0 - 38.8)	9.3 (8.0 - 10.5)
Median LOS (discharge)	3.7 (3.4 - 4.0)	2.5 (1.8 - 3.2)	4.6 (4.3 - 4.9)
**Aug-20**			
HFR	11.0% (7.7 - 15.7%)	4.9% (1.9 - 12.8%)	15.6% (12.6 - 19.3%)
Median LOS (death)	11.5 (11.0 - 13.0)	7.5 (4.7 - 8.6)	10.4 (9.8 - 11.3)
Median LOS (discharge)	3.1 (2.8 - 3.5)	2.3 (1.8 - 2.9)	3.3 (3.0 - 3.5)
**Sep-20**			
HFR	13.5% (11.4 - 16.1%)	7.1% (4.4 - 11.6%)	17.5% (16.1 - 19.1%)
Median LOS (death)	9.9 (9.6 - 10.3)	11.0 (9.6 - 17.3)	8.5 (8.0 - 8.9)
Median LOS (discharge)	3.3 (3.1 - 3.5)	3.8 (3.4 - 4.2)	4.4 (4.3 - 4.6)
**Oct-20**			
HFR	13.8% (12.3 - 15.5%)	10.8% (8.3 - 14.0%)	20.8% (20.1 - 21.6%)
Median LOS (death)	9.0 (8.5 - 9.5)	8.4 (7.0 - 9.0)	8.4 (8.2 - 8.6)
Median LOS (discharge)	3.6 (3.4 - 3.7)	3.7 (3.4 - 4.0)	4.8 (4.7 - 4.9)
**Nov-20**			
HFR	14.8% (13.5 - 16.2%)	11.7% (9.6 - 14.3%)	22.5% (21.9 - 23.1%)
Median LOS (death)	8.2 (7.9 - 8.5)	9.9 (9.5 - 11.4)	8.4 (8.3 - 8.5)
Median LOS (discharge)	3.5 (3.4 - 3.6)	4.5 (4.2 - 4.7)	5.2 (5.1 - 5.2)
**Dec-20**			
HFR	17.5% (16.3 - 18.7%)	14.3% (12.7 - 16.0%)	24.8% (24.2 - 25.4%)
Median LOS (death)	9.9 (9.6 - 10.2)	10.2 (9.6 - 11.0)	8.2 (8.1 - 8.3)
Median LOS (discharge)	3.9 (3.8 - 4.0)	4.3 (4.1 - 4.4)	5.5 (5.4 - 5.5)
**Jan-21**			

Supplementary table 5 continued from previous page

HFR	18.3% (17.4 - 19.2%)	15.9% (14.7 - 17.1%)	24.8% (24.4 - 25.2%)
Median LOS (death)	9.4 (9.2 - 9.6)	8.7 (8.4 - 9.0)	7.3 (7.2 - 7.3)
Median LOS (discharge)	4.1 (4.0 - 4.2)	4.3 (4.2 - 4.4)	5.2 (5.2 - 5.3)
**Feb-21**			
HFR	14.4% (13.0 - 15.9%)	13.3% (11.2 - 15.9%)	19.1% (18.5 - 19.8%)
Median LOS (death)	9.1 (8.8 - 9.5)	9.0 (8.4 - 9.9)	7.5 (7.4 - 7.7)
Median LOS (discharge)	3.6 (3.5 - 3.7)	4.0 (3.9 - 4.2)	4.9 (4.8 - 4.9)
**Mar-21**			
HFR	8.7% (7.0 - 11.0%)	5.4% (3.1 - 9.3%)	11.6% (10.7 - 12.7%)
Median LOS (death)	11.0 (10.5 - 12.1)	9.3 (5.8 - 14.3)	8.3 (8.0 - 8.7)
Median LOS (discharge)	2.8 (2.6 - 3.0)	2.9 (2.6 - 3.3)	3.9 (3.8 - 4.0)
**Apr-21**			
HFR	7.9% (5.6 - 11.2%)	4.9% (1.6 - 14.8%)	10.1% (8.5 - 11.9%)
Median LOS (death)	6.2 (6.0 - 6.7)	4.0 (1.8 - 8.8)	9.2 (8.8 - 9.7)
Median LOS (discharge)	2.7 (2.4 - 2.9)	1.9 (1.4 - 2.4)	3.5 (3.4 - 3.7)
**May-21**			
HFR	9.8% (7.3 - 13.2%)	7.0% (3.3 - 15.2%)	9.2% (7.3 - 11.7%)
Median LOS (death)	10.3 (7.9 - 12.2)	11.0 (5.3 - 14.4)	9.2 (8.7 - 10.2)
Median LOS (discharge)	3.0 (2.8 - 3.3)	2.3 (1.8 - 3.0)	2.9 (2.8 - 3.2)
**Jun-21**			
HFR	8.6% (6.7 - 11.2%)	6.2% (4.1 - 9.5%)	8.6% (7.5 - 9.8%)
Median LOS (death)	11.7 (10.8 - 12.7)	10.5 (8.1 - 14.0)	10.2 (9.8 - 10.5)
Median LOS (discharge)	2.7 (2.5 - 2.9)	3.5 (3.0 - 3.9)	2.6 (2.5 - 2.7)
**Jul-21**			
HFR	8.4% (7.0 - 10.1%)	8.0% (6.5 - 9.9%)	9.3% (8.8 - 9.9%)
Median LOS (death)	12.1 (11.5 - 12.9)	11.8 (11.3 - 12.3)	9.7 (9.5 - 9.9)
Median LOS (discharge)	2.7 (2.6 - 2.9)	3.9 (3.6 - 4.1)	3.0 (2.9 - 3.0)
**Aug-21**			
HFR	11.4% (9.9 - 13.1%)	8.9% (7.4 - 10.7%)	11.4% (10.8 - 12.0%)
Median LOS (death)	9.6 (9.1 - 10.2)	10.8 (10.2 - 11.4)	9.2 (9.0 - 9.5)
Median LOS (discharge)	2.9 (2.8 - 3.0)	3.9 (3.8 - 4.1)	3.3 (3.2 - 3.3)
**Sep-21**			
HFR	9.3% (7.7 - 11.2%)	8.3% (6.4 - 10.6%)	11.5% (10.8 - 12.1%)
Median LOS (death)	8.6 (8.1 - 9.2)	8.8 (8.4 - 9.9)	7.5 (7.3 - 7.7)
Median LOS (discharge)	3.0 (2.8 - 3.2)	4.0 (3.8 - 4.2)	3.7 (3.6 - 3.7)

Supplementary table 6: Hospitalised fatality risk and median length of stay (days) in hospital prior to death or discharge by month of hospital admission and region of residence (unadjusted for other covariates). Figures in brackets represent 95% confidence intervals.

**Month of hospital admission**	**London**	**East Midlands**	**East of England**
**Mar-20**			
HFR	40.8% (39.2 - 42.4%)	33.9% (30.9 - 37.1%)	42.5% (39.5 - 45.6%)
Median LOS (death)	5.8 (5.7 - 6.0)	6.2 (5.9 - 6.5)	5.8 (5.6 - 6.1)
Median LOS (discharge)	6.1 (5.9 - 6.2)	6.0 (5.8 - 6.3)	5.6 (5.4 - 5.8)
**Apr-20**			
HFR	35.3% (34.0 - 36.7%)	34.9% (32.7 - 37.1%)	41.9% (40.1 - 43.8%)
Median LOS (death)	4.9 (4.8 - 5.0)	5.0 (4.9 - 5.2)	5.3 (5.1 - 5.4)
Median LOS (discharge)	5.4 (5.3 - 5.5)	5.5 (5.4 - 5.7)	5.9 (5.8 - 6.1)
**May-20**			
HFR	19.4% (16.3 - 23.1%)	28.4% (25.4 - 31.8%)	29.2% (26.4 - 32.2%)
Median LOS (death)	8.6 (7.9 - 9.3)	7.0 (6.6 - 7.4)	5.9 (5.7 - 6.3)
Median LOS (discharge)	4.9 (4.5 - 5.2)	6.6 (6.3 - 6.9)	5.8 (5.5 - 6.0)
**Jun-20**			
HFR	7.0% (4.3 - 11.3%)	21.3% (17.5 - 25.8%)	16.9% (13.3 - 21.4%)
Median LOS (death)	10.5 (10.3 - 12.2)	7.8 (7.5 - 8.5)	8.8 (8.4 - 10.0)
Median LOS (discharge)	3.3 (2.8 - 4.0)	5.5 (5.2 - 5.8)	4.6 (4.2 - 5.1)
**Jul-20**			
HFR	6.3% (3.2 - 12.2%)	10.6% (6.5 - 17.0%)	8.1% (4.5 - 14.6%)
Median LOS (death)	13.5 (8.6 - 15.8)	7.7 (4.9 - 9.6)	5.0 (2.3 - 10.6)
Median LOS (discharge)	4.2 (3.0 - 5.1)	4.2 (3.7 - 4.7)	4.8 (4.1 - 5.4)
**Aug-20**			
HFR	7.1% (3.6 - 13.8%)	12.8% (7.0 - 23.6%)	5.7% (1.9 - 17.2%)
Median LOS (death)	12.0 (6.6 - 18.8)	12.0 (7.5 - 14.9)	3.0 (1.5 - 3.8)
Median LOS (discharge)	2.9 (2.3 - 3.4)	5.4 (4.9 - 5.8)	2.7 (1.5 - 3.6)
**Sep-20**			
HFR	9.6% (7.3 - 12.7%)	10.9% (7.5 - 15.7%)	13.8% (9.6 - 19.8%)
Median LOS (death)	10.7 (10.4 - 12.4)	10.2 (8.3 - 10.9)	11.0 (7.6 - 12.8)
Median LOS (discharge)	4.4 (4.1 - 4.7)	4.0 (3.7 - 4.4)	3.0 (2.7 - 3.4)
**Oct-20**			
HFR	12.9% (11.3 - 14.8%)	20.1% (18.0 - 22.4%)	23.1% (20.3 - 26.3%)
Median LOS (death)	10.7 (9.5 - 11.5)	8.4 (8.1 - 8.8)	8.3 (7.6 - 8.8)
Median LOS (discharge)	4.4 (4.3 - 4.6)	4.5 (4.4 - 4.7)	4.4 (4.2 - 4.6)
**Nov-20**			
HFR	15.7% (14.3 - 17.3%)	21.9% (20.3 - 23.6%)	20.3% (18.4 - 22.5%)
Median LOS (death)	9.5 (9.1 - 9.9)	8.6 (8.3 - 8.9)	9.3 (8.9 - 9.7)
Median LOS (discharge)	4.9 (4.8 - 5.0)	4.9 (4.7 - 5.0)	4.7 (4.6 - 4.9)
**Dec-20**			
HFR	18.7% (17.8 - 19.6%)	26.8% (25.1 - 28.7%)	25.3% (23.9 - 26.7%)
Median LOS (death)	9.2 (9.0 - 9.4)	9.1 (8.8 - 9.3)	8.3 (8.0 - 8.5)
Median LOS (discharge)	4.6 (4.6 - 4.7)	5.1 (5.0 - 5.3)	5.1 (5.0 - 5.2)
**Jan-21**			
HFR	19.8% (19.1 - 20.5%)	24.5% (23.3 - 25.8%)	26.5% (25.5 - 27.5%)
Median LOS (death)	7.9 (7.7 - 8.0)	7.1 (6.9 - 7.3)	6.8 (6.6 - 6.9)
Median LOS (discharge)	4.5 (4.4 - 4.5)	5.2 (5.1 - 5.3)	5.0 (4.9 - 5.0)
**Feb-21**			

Supplementary table 6 continued from previous page

HFR	14.2% (12.9 - 15.7%)	19.7% (18.0 - 21.6%)	19.8% (18.2 - 21.7%)
Median LOS (death)	8.8 (8.5 - 9.3)	7.7 (7.3 - 8.0)	7.2 (6.9 - 7.6)
Median LOS (discharge)	4.1 (4.0 - 4.2)	5.2 (5.1 - 5.4)	4.7 (4.6 - 4.8)
**Mar-21**			
HFR	8.2% (6.3 - 10.7%)	11.6% (9.4 - 14.3%)	13.7% (10.9 - 17.3%)
Median LOS (death)	12.2 (9.8 - 13.5)	10.0 (9.1 - 11.2)	10.5 (10.0 - 11.3)
Median LOS (discharge)	3.5 (3.3 - 3.7)	4.1 (3.8 - 4.3)	3.8 (3.5 - 4.0)
**Apr-21**			
HFR	6.5% (3.9 - 10.8%)	10.6% (7.0 - 16.1%)	7.9% (4.8 - 13.0%)
Median LOS (death)	9.3 (4.4 - 12.5)	6.7 (3.8 - 8.4)	10.3 (5.8 - 11.7)
Median LOS (discharge)	2.4 (2.1 - 2.8)	4.1 (3.6 - 4.5)	4.2 (3.7 - 4.8)
**May-21**			
HFR	7.2% (4.5 - 11.5%)	5.7% (2.6 - 12.5%)	6.7% (3.4 - 13.1%)
Median LOS (death)	17.0 (9.6 - 20.7)	13.0 (2.4 - 13.4)	16.5 (4.6 - 19.4)
Median LOS (discharge)	3.7 (3.3 - 4.0)	2.8 (2.4 - 3.3)	2.9 (2.5 - 3.3)
**Jun-21**			
HFR	8.6% (6.6 - 11.2%)	8.9% (5.7 - 13.8%)	6.5% (3.9 - 10.8%)
Median LOS (death)	11.3 (10.7 - 12.9)	8.0 (7.8 - 12.2)	8.5 (5.9 - 11.0)
Median LOS (discharge)	2.9 (2.7 - 3.1)	2.7 (2.4 - 2.9)	2.6 (2.3 - 2.9)
**Jul-21**			
HFR	7.7% (6.7 - 9.0%)	8.4% (6.8 - 10.3%)	8.3% (6.8 - 10.2%)
Median LOS (death)	11.8 (11.4 - 12.4)	9.5 (8.9 - 10.4)	11.5 (10.6 - 12.2)
Median LOS (discharge)	3.3 (3.2 - 3.5)	3.4 (3.2 - 3.6)	2.5 (2.4 - 2.7)
**Aug-21**			
HFR	9.5% (8.4 - 10.8%)	11.5% (10.0 - 13.3%)	10.1% (8.6 - 11.9%)
Median LOS (death)	10.8 (10.5 - 11.2)	9.4 (9.1 - 9.9)	8.6 (8.3 - 9.2)
Median LOS (discharge)	3.6 (3.5 - 3.7)	3.6 (3.4 - 3.7)	3.0 (2.9 - 3.2)
**Sep-21**			
HFR	9.4% (8.1 - 11.0%)	12.2% (10.5 - 14.2%)	11.0% (9.2 - 13.2%)
Median LOS (death)	8.9 (8.5 - 9.4)	8.2 (7.7 - 8.7)	6.7 (6.4 - 7.1)
Median LOS (discharge)	3.9 (3.8 - 4)	3.7 (3.6 - 3.9)	3.4 (3.2 - 3.5)

Supplementary table 6 continued

Month of hospital admission	North East	North West	South East
**Mar-20**			
HFR	38.2% (32.3 - 45.2%)	41.4% (38.9 - 44.0%)	38.2% (35.7 - 41.0%)
Median LOS (death)	5.3 (5.0 - 5.7)	5.3 (5.1 - 5.6)	5.3 (5.0 - 5.5)
Median LOS (discharge)	6.6 (6.2 - 7.1)	6.3 (6.1 - 6.5)	6.2 (6.0 - 6.4)
**Apr-20**			
HFR	36.5% (34.1 - 39.1%)	38.8% (37.3 - 40.3%)	34.7% (33.0 - 36.5%)
Median LOS (death)	4.9 (4.7 - 5.1)	5.3 (5.2 - 5.5)	5.5 (5.3 - 5.6)
Median LOS (discharge)	5.9 (5.8 - 6.1)	6.1 (6.0 - 6.2)	5.9 (5.8 - 6.1)
**May-20**			
HFR	39.4% (34.7 - 44.7%)	30.7% (28.3 - 33.3%)	28.6% (25.5 - 31.9%)
Median LOS (death)	7.2 (6.7 - 7.7)	5.9 (5.6 - 6.2)	6.4 (6.0 - 6.8)
Median LOS (discharge)	4.3 (3.9 - 4.7)	5.7 (5.5 - 6.0)	5.7 (5.4 - 5.9)
**Jun-20**			
HFR	17.8% (11.3 - 28.1%)	24.8% (21.2 - 28.9%)	21.8% (17.4 - 27.1%)

Supplementary table 6 continued from previous page

Median LOS (death)	7.0 (4.8 - 14.0)	6.7 (6.3 - 7.5)	7.0 (6.6 - 7.6)
Median LOS (discharge)	4.7 (3.6 - 6.6)	5.3 (4.9 - 5.7)	5.4 (4.9 - 5.8)
**Jul-20**			
HFR	10.0% (2.7 - 37.2%)	17.1% (12.6 - 23.4%)	21.1% (14.9 - 29.8%)
Median LOS (death)	9.5 (2.6 - 13.3)	10.3 (10.1 - 10.8)	7.0 (6.3 - 9.9)
Median LOS (discharge)	5.5 (3.6 - 8.1)	4.0 (3.5 - 4.5)	3.8 (3.4 - 4.7)
**Aug-20**			
HFR	17.6% (6.3 - 49.3%)	16.1% (12.1 - 21.4%)	14.0% (7.0 - 27.8%)
Median LOS (death)	6.0 (1.4 - 7.2)	7.8 (7.5 - 9.4)	7.0 (3.9 - 7.8)
Median LOS (discharge)	2.5 (1.3 - 4.9)	2.8 (2.5 - 3.1)	5.0 (3.8 - 6.0)
**Sep-20**			
HFR	19.8% (16.3 - 24.1%)	19.3% (17.2 - 21.7%)	14.7% (10.3 - 20.9%)
Median LOS (death)	6.6 (5.6 - 7.3)	8.7 (8.3 - 9.2)	7.5 (5.2 - 10.7)
Median LOS (discharge)	3.6 (3.3 - 3.9)	4.9 (4.8 - 5.1)	4.3 (3.9 - 4.7)
**Oct-20**			
HFR	19.7% (17.7 - 22.0%)	21.2% (20.0 - 22.4%)	15.1% (12.8 - 17.8%)
Median LOS (death)	7.4 (7.0 - 7.8)	7.8 (7.6 - 8.1)	8.5 (8.0 - 9.2)
Median LOS (discharge)	4.3 (4.1 - 4.5)	4.7 (4.6 - 4.8)	4.3 (4.1 - 4.5)
**Nov-20**			
HFR	20.0% (18.2 - 22.0%)	23.7% (22.5 - 25.1%)	19.8% (18.2 - 21.6%)
Median LOS (death)	7.0 (6.8 - 7.4)	8.1 (7.9 - 8.3)	8.7 (8.4 - 9.0)
Median LOS (discharge)	4.6 (4.4 - 4.8)	4.9 (4.9 - 5.1)	5.1 (4.9 - 5.2)
**Dec-20**			
HFR	22.7% (20.5 - 25.1%)	24.2% (22.7 - 25.8%)	22.1% (21.0 - 23.2%)
Median LOS (death)	7.4 (7.1 - 7.9)	7.7 (7.5 - 8.0)	9.0 (8.8 - 9.3)
Median LOS (discharge)	5.3 (5.1 - 5.4)	5.2 (5.1 - 5.3)	5.3 (5.2 - 5.3)
**Jan-21**			
HFR	22.2% (20.6 - 23.9%)	24.4% (23.4 - 25.4%)	23.5% (22.6 - 24.3%)
Median LOS (death)	8.1 (7.8 - 8.4)	7.9 (7.7 - 8.1)	7.7 (7.5 - 7.9)
Median LOS (discharge)	4.7 (4.6 - 4.8)	5.1 (5.0 - 5.2)	5.0 (4.9 - 5.0)
**Feb-21**			
HFR	15.3% (13.4 - 17.6%)	19.7% (18.4 - 21.2%)	17.0% (15.6 - 18.6%)
Median LOS (death)	7.0 (6.4 - 7.6)	7.6 (7.4 - 7.9)	8.5 (8.1 - 8.9)
Median LOS (discharge)	4.2 (4.0 - 4.4)	4.6 (4.5 - 4.8)	4.8 (4.7 - 4.9)
**Mar-21**			
HFR	7.0% (4.9 - 10.1%)	11.5% (9.7 - 13.6%)	9.7% (7.5 - 12.6%)
Median LOS (death)	10.2 (10.0 - 11.0)	7.3 (6.7 - 7.9)	8.0 (7.4 - 9.2)
Median LOS (discharge)	3.0 (2.8 - 3.4)	3.7 (3.5 - 3.9)	4.0 (3.8 - 4.3)
**Apr-21**			
HFR	5.1% (2.2 - 12.0%)	10.3% (7.3 - 14.4%)	3.4% (1.6 - 7.6%)
Median LOS (death)	7.0 (5.5 - 10.4)	6.3 (5.9 - 7.4)	10.0 (4.1 - 10.4)
Median LOS (discharge)	1.5 (1.2 - 1.9)	3.4 (3.1 - 3.6)	3.6 (3.2 - 4.0)
**May-21**			
HFR	11.8% (5.6 - 25.1%)	10.1% (7.3 - 14.0%)	5.2% (2.0 - 13.5%)
Median LOS (death)	3.5 (2.1 - 4.1)	9.0 (8.2 - 10.2)	14.5 (6.9 - 17.3)
Median LOS (discharge)	3.2 (2.9 - 3.5)	2.9 (2.6 - 3.1)	2.7 (2.3 - 3.2)
**Jun-21**			
HFR	5.2% (3.0 - 8.8%)	9.9% (8.3 - 11.7%)	5.1% (2.8 - 9.0%)
Median LOS (death)	7.0 (5.3 - 10.5)	10.8 (10.4 - 11.4)	8.0 (3.6 - 9.4)
Median LOS (discharge)	2.2 (1.9 - 2.4)	2.5 (2.3 - 2.6)	2.9 (2.5 - 3.2)

Supplementary table 6 continued from previous page

**Jul-21**			
HFR	9.0% (7.6 - 10.6%)	11.6% (10.4 - 13.0%)	8.7% (7.3 - 10.4%)
Median LOS (death)	8.6 (7.9 - 9.2)	9.5 (9.2 - 9.9)	10.1 (9.8 - 10.8)
Median LOS (discharge)	2.6 (2.5 - 2.7)	3.4 (3.3 - 3.5)	2.8 (2.6 - 2.9)
**Aug-21**			
HFR	10.5% (8.7 - 12.6%)	11.9% (10.7 - 13.4%)	10.0% (8.6 - 11.6%)
Median LOS (death)	9.5 (8.3 - 10.4)	10.0 (9.5 - 10.4)	9.3 (8.7 - 10.3)
Median LOS (discharge)	2.7 (2.6 - 2.9)	3.1 (3.0 - 3.3)	3.0 (2.9 - 3.2)
**Sep-21**			
HFR	12.9% (10.9 - 15.3%)	10.5% (9.2 - 12.0%)	9.1% (7.7 - 10.9%)
Median LOS (death)	7.5 (7.2 - 7.8)	7.2 (6.9 - 7.6)	8.6 (8.3 - 9.0)
Median LOS (discharge)	3.3 (3.2 - 3.5)	3.6 (3.4 - 3.7)	3.5 (3.3 - 3.7)

Supplementary table 6 continued

**Month of hospital admission**	**South West**	**West Midlands**	**Yorkshire and Humber**
**Mar-20**			
HFR	37.6% (33.8 - 41.8%)	42.0% (39.4 - 44.7%)	45.6% (41.8 - 49.6%)
Median LOS (death)	5.5 (5.2 - 5.9)	5.1 (4.9 - 5.3)	5.5 (5.2 - 5.9)
Median LOS (discharge)	5.2 (4.9 - 5.6)	4.7 (4.5 - 4.9)	6.3 (6.0 - 6.5)
**Apr-20**			
HFR	30.4% (28.1 - 33.0%)	39.0% (37.1 - 41.0%)	41.5% (39.6 - 43.6%)
Median LOS (death)	5.3 (5.1 - 5.5)	5.3 (5.1 - 5.5)	5.4 (5.2 - 5.6)
Median LOS (discharge)	5.9 (5.7 - 6.1)	6.3 (6.1 - 6.4)	5.9 (5.8 - 6.1)
**May-20**			
HFR	24.5% (20.6 - 29.0%)	30.6% (27.4 - 34.1%)	34.7% (31.7 - 38.0%)
Median LOS (death)	6.1 (5.5 - 6.9)	6.6 (6.3 - 7.0)	6.8 (6.5 - 7.2)
Median LOS (discharge)	5.2 (4.7 - 5.8)	6.1 (5.8 - 6.4)	4.7 (4.5 - 4.9)
**Jun-20**			
HFR	12.3% (7.4 - 20.4%)	20.7% (16.3 - 26.2%)	26.9% (22.3 - 32.3%)
Median LOS (death)	10.0 (3.8 - 13.1)	7.4 (6.4 - 8.2)	8.0 (7.5 - 8.8)
Median LOS (discharge)	3.9 (3.3 - 4.8)	6.0 (5.5 - 6.6)	4.0 (3.7 - 4.4)
**Jul-20**			
HFR	5.8% (1.5 - 22.2%)	10.7% (6.3 - 18.2%)	18.7% (12.9 - 27.0%)
Median LOS (death)	3.0 (1.4 - 3.9)	12.5 (7.6 - 14.6)	12.7 (12.3 - 13.6)
Median LOS (discharge)	3.6 (2.7 - 4.2)	3.5 (3.1 - 3.9)	3.6 (3.0 - 4.2)
**Aug-20**			
HFR	9.6% (3.8 - 24.4%)	8.5% (4.7 - 15.4%)	15.1% (10.2 - 22.4%)
Median LOS (death)	9.3 (6.1 - 9.6)	12.5 (6.6 - 13.5)	14.5 (14.1 - 15.5)
Median LOS (discharge)	3.0 (2.1 - 3.7)	2.8 (2.3 - 3.3)	3.1 (2.8 - 3.5)
**Sep-20**			
HFR	7.3% (3.4 - 15.7%)	13.7% (11.2 - 16.8%)	14.8% (11.9 - 18.3%)
Median LOS (death)	9.0 (1.8 - 14.2)	9.6 (8.6 - 10.3)	9.5 (8.9 - 10.4)
Median LOS (discharge)	5.3 (4.8 - 5.7)	3.8 (3.5 - 4.0)	2.8 (2.6 - 3.1)
**Oct-20**			
HFR	14.0% (11.6 - 16.8%)	19.1% (17.3 - 21.1%)	19.4% (17.8 - 21.1%)
Median LOS (death)	9.0 (8.7 - 9.4)	9.5 (9.2 - 9.9)	9.2 (8.7 - 9.7)
Median LOS (discharge)	4.8 (4.6 - 5.1)	4.4 (4.2 - 4.5)	4.4 (4.3 - 4.5)
**Nov-20**			

Supplementary table 6 continued from previous page

HFR	16.2% (14.5 - 18.0%)	21.9% (20.5 - 23.4%)	22.1% (20.7 - 23.6%)
Median LOS (death)	8.7 (8.5 - 8.9)	8.5 (8.2 - 8.8)	8.2 (7.9 - 8.5)
Median LOS (discharge)	4.2 (4.0 - 4.4)	5.1 (5.0 - 5.3)	4.7 (4.6 - 4.8)
**Dec-20**			
HFR	17.5% (15.8 - 19.4%)	24.2% (22.7 - 25.8%)	26.7% (24.8 - 28.7%)
Median LOS (death)	9.0 (8.7 - 9.4)	7.5 (7.3 - 7.8)	7.4 (7.1 - 7.8)
Median LOS (discharge)	5.4 (5.2 - 5.6)	5.5 (5.4 - 5.6)	4.8 (4.7 - 5.0)
**Jan-21**			
HFR	19.2% (18.1 - 20.4%)	23.7% (22.7 - 24.8%)	21.8% (20.4 - 23.2%)
Median LOS (death)	6.9 (6.7 - 7.1)	7.7 (7.5 - 7.8)	8.2 (7.9 - 8.5)
Median LOS (discharge)	5.2 (5.1 - 5.3)	5.2 (5.2 - 5.3)	4.8 (4.7 - 5.0)
**Feb-21**			
HFR	15.9% (14.0 - 17.9%)	19.0% (17.5 - 20.5%)	17.6% (15.9 - 19.5%)
Median LOS (death)	6.4 (6.2 - 6.7)	8.4 (8.0 - 8.8)	7.6 (7.4 - 8.0)
Median LOS (discharge)	4.8 (4.6 - 4.9)	4.6 (4.5 - 4.7)	4.4 (4.3 - 4.6)
**Mar-21**			
HFR	8.0% (5.5 - 11.5%)	12.8% (10.5 - 15.6%)	9.8% (8.0 - 11.9%)
Median LOS (death)	6.8 (6.7 - 7.9)	8.1 (7.5 - 8.6)	8.4 (8.1 - 8.9)
Median LOS (discharge)	3.8 (3.5 - 4.1)	3.6 (3.4 - 3.9)	3.3 (3.1 - 3.5)
**Apr-21**			
HFR	14.9% (9.0 - 24.5%)	9.9% (6.6 - 15.1%)	9.5% (7.0 - 12.9%)
Median LOS (death)	12.0 (11.9 - 13.2)	9.0 (7.4 - 10.8)	8.3 (7.9 - 9.4)
Median LOS (discharge)	4.4 (4.1 - 4.7)	2.8 (2.5 - 3.1)	3.0 (2.8 - 3.2)
**May-21**			
HFR	10.1% (4.0 - 25.6%)	9.2% (5.7 - 14.9%)	10.3% (6.8 - 15.5%)
Median LOS (death)	10.5 (6.7 - 13.6)	9.7 (8.2 - 11.6)	8.0 (4.0 - 9.0)
Median LOS (discharge)	1.0 (0.9 - 1.7)	2.8 (2.4 - 3.3)	1.9 (1.7 - 2.2)
**Jun-21**			
HFR	3.7% (1.8 - 7.6%)	8.6% (6.2 - 12.1%)	7.3% (5.0 - 10.6%)
Median LOS (death)	10.0 (1.8 - 10.8)	9.0 (6.7 - 11.0)	11.3 (9.9 - 12.2)
Median LOS (discharge)	2.6 (2.1 - 3.0)	3.0 (2.7 - 3.3)	3.1 (2.8 - 3.4)
**Jul-21**			
HFR	5.8% (4.5 - 7.4%)	8.3% (7.1 - 9.8%)	8.4% (7.2 - 9.9%)
Median LOS (death)	8.6 (7.7 - 9.3)	10.7 (10.2 - 11.6)	9.3 (8.6 - 9.9)
Median LOS (discharge)	2.7 (2.6 - 2.9)	3.0 (2.9 - 3.1)	3.2 (3.0 - 3.3)
**Aug-21**			
HFR	9.7% (8.3 - 11.4%)	10.6% (9.2 - 12.2%)	12.1% (10.7 - 13.7%)
Median LOS (death)	7.6 (7.1 - 8.6)	10.0 (9.6 - 10.6)	9.3 (8.9 - 9.8)
Median LOS (discharge)	3.1 (2.9 - 3.2)	3.3 (3.2 - 3.4)	3.2 (3.1 - 3.4)
**Sep-21**			
HFR	9.9% (8.4 - 11.8%)	11.0% (9.5 - 12.7%)	9.9% (8.5 - 11.6%)
Median LOS (death)	6.8 (6.5 - 7.1)	7.4 (7.0 - 8.0)	7.4 (7.1 - 7.9)
Median LOS (discharge)	2.9 (2.8 - 3.1)	3.8 (3.7 - 4.0)	3.6 (3.5 - 3.8)

Supplementary table 7: Hospitalised fatality risk and median length of stay (days) in hospital prior to death or discharge by month of hospital admission and Charleson Comorbidity Index (unadjusted for other covariates). Figures in brackets represent 95% confidence intervals.

Month of hospital admission	0	1-2	3-4	5+
**Mar-20**				
HFR	17.4% (16.1 - 18.8%)	36.3% (35.0 - 37.8%)	53.4% (51.1 - 55.8%)	53.3% (50.0 - 56.8%)
Median LOS (death)	6.0 (5.8 - 6.3)	5.6 (5.5 - 5.8)	4.9 (4.8 - 5.1)	5.0 (4.8 - 5.3)
Median LOS (discharge)	4.4 (4.3 - 4.5)	6.4 (6.3 - 6.5)	8.2 (8.0 - 8.4)	8.3 (8.0 - 8.6)
**Apr-20**				
HFR	15.2% (14.4 - 16.2%)	34.4% (33.5 - 35.4%)	48.1% (46.6 - 49.6%)	52.4% (50.2 - 54.7%)
Median LOS (death)	5.6 (5.5 - 5.7)	5.1 (5.0 - 5.2)	4.9 (4.8 - 5.0)	4.6 (4.5 - 4.8)
Median LOS (discharge)	4.3 (4.2 - 4.3)	6.2 (6.1 - 6.2)	7.9 (7.8 - 8.0)	8.2 (8.0 - 8.4)
**May-20**				
HFR	7.3% (6.2 - 8.7%)	26.0% (24.3 - 27.7%)	36.5% (34.0 - 39.2%)	45.1% (41.6 - 49.0%)
Median LOS (death)	6.6 (6.3 - 7.1)	6.0 (5.8 - 6.2)	6.0 (5.8 - 6.3)	6.6 (6.2 - 7.0)
Median LOS (discharge)	2.8 (2.7 - 2.9)	6.3 (6.2 - 6.4)	8.3 (8.1 - 8.6)	7.4 (7.0 - 7.8)
**Jun-20**				
HFR	3.9% (2.8 - 5.6%)	18.3% (16.0 - 21.0%)	28.7% (25.0 - 33.0%)	36.6% (31.2 - 42.8%)
Median LOS (death)	8.5 (7.3 - 9.2)	7.4 (7.0 - 7.8)	6.9 (6.5 - 7.4)	6.9 (6.4 - 7.6)
Median LOS (discharge)	2.3 (2.1 - 2.5)	5.3 (5.1 - 5.6)	8.3 (7.9 - 8.7)	7.6 (7.2 - 7.9)
**Jul-20**				
HFR	3.3% (1.9 - 5.7%)	12.6% (9.6 - 16.6%)	20.1% (14.6 - 27.7%)	25.8% (19.1 - 34.8%)
Median LOS (death)	9.5 (5.4 - 11.6)	8.0 (7.5 - 9.5)	9.0 (8.5 - 10.1)	12.5 (9.4 - 14.7)
Median LOS (discharge)	1.9 (1.8 - 2.2)	4.4 (4.1 - 4.8)	7.0 (6.3 - 7.7)	9.0 (8.0 - 9.7)
**Aug-20**				
HFR	2.6% (1.4 - 4.8%)	11.7% (8.5 - 15.9%)	26.9% (19.7 - 36.7%)	34.7% (23.6 - 50.9%)
Median LOS (death)	12.0 (12.2 - 13.2)	7.8 (7.6 - 8.6)	10.7 (8.6 - 11.8)	8.0 (5.7 - 10.3)
Median LOS (discharge)	1.6 (1.4 - 1.8)	4.7 (4.2 - 5.1)	7.0 (6.3 - 8.7)	6.7 (5.5 - 8.1)
**Sep-20**				
HFR	4.3% (3.4 - 5.4%)	14.6% (12.9 - 16.4%)	31.7% (28.0 - 36.0%)	45.7% (39.7 - 52.6%)
Median LOS (death)	11.5 (10.6 - 12.6)	9.7 (9.3 - 10.1)	8.9 (8.0 - 9.4)	5.8 (4.9 - 6.7)
Median LOS (discharge)	2.6 (2.4 - 2.7)	5.0 (4.8 - 5.2)	7.2 (6.8 - 7.6)	6.8 (6.5 - 7.1)
**Oct-20**				
HFR	5.4% (4.8 - 6.0%)	19.4% (18.4 - 20.4%)	34.3% (32.3 - 36.3%)	41.7% (38.8 - 44.9%)
Median LOS (death)	11.2 (10.9 - 11.6)	8.9 (8.7 - 9.1)	7.4 (7.1 - 7.6)	7.2 (6.9 - 7.6)
Median LOS (discharge)	2.9 (2.9 - 3.0)	5.2 (5.1 - 5.3)	7.2 (7.0 - 7.5)	8.1 (7.8 - 8.3)
**Nov-20**				
HFR	6.1% (5.6 - 6.7%)	20.0% (19.2 - 20.9%)	36.0% (34.5 - 37.6%)	40.9% (38.7 - 43.3%)
Median LOS (death)	10.9 (10.5 - 11.2)	8.6 (8.4 - 8.8)	7.7 (7.5 - 7.9)	7.5 (7.2 - 7.7)
Median LOS (discharge)	3.1 (3.0 - 3.1)	5.6 (5.5 - 5.7)	7.9 (7.7 - 8.0)	8.0 (7.8 - 8.2)
**Dec-20**				
HFR	6.7% (6.2 - 7.2%)	22.4% (21.6 - 23.1%)	38.7% (37.3 - 40.2%)	47.3% (45.3 - 49.4%)
Median LOS (death)	10.6 (10.3 - 11.0)	8.7 (8.5 - 8.8)	7.9 (7.7 - 8.1)	7.0 (6.8 - 7.2)
Median LOS (discharge)	3.4 (3.3 - 3.4)	6.0 (5.9 - 6.1)	8.4 (8.2 - 8.5)	8.9 (8.7 - 9.0)
**Jan-21**				
HFR	7.9% (7.5 - 8.2%)	22.8% (22.3 - 23.3%)	39.6% (38.5 - 40.6%)	46.3% (44.8 - 47.9%)
Median LOS (death)	9.7 (9.5 - 9.9)	7.5 (7.4 - 7.6)	6.8 (6.7 - 6.9)	6.6 (6.5 - 6.8)
Median LOS (discharge)	3.6 (3.6 - 3.6)	5.7 (5.6 - 5.7)	7.9 (7.8 - 8.1)	8.0 (7.9 - 8.2)

Supplementary table 7 continued from previous page

**Feb-21**				
HFR	5.5% (5.0 - 6.1%)	17.4% (16.5 - 18.2%)	32.6% (30.9 - 34.5%)	39.1% (36.6 - 41.7%)
Median LOS (death)	10.3 (10.0 - 10.7)	8.0 (7.8 - 8.3)	7.1 (6.9 - 7.4)	6.7 (6.5 - 7.0)
Median LOS (discharge)	3.0 (3.0 - 3.1)	5.4 (5.3 - 5.5)	7.6 (7.4 - 7.8)	7.9 (7.7 - 8.2)
**Mar-21**				
HFR	1.9% (1.4 - 2.5%)	10.8% (9.5 - 12.2%)	24.3% (21.3 - 27.8%)	36.0% (31.3 - 41.3%)
Median LOS (death)	12.4 (12.1 - 13.8)	8.9 (8.6 - 9.4)	7.3 (7.0 - 7.7)	8.1 (7.5 - 9.5)
Median LOS (discharge)	2.4 (2.3 - 2.5)	4.5 (4.4 - 4.7)	6.8 (6.4 - 7.1)	7.1 (6.8 - 7.6)
**Apr-21**				
HFR	1.1% (0.6 - 2.0%)	9.1% (7.0 - 11.8%)	21.8% (17.1 - 27.9%)	26.9% (20.3 - 35.5%)
Median LOS (death)	8.5 (8.5 - 8.9)	9.3 (7.3 - 10.5)	7.4 (6.1 - 8.5)	9.3 (9.1 - 10.0)
Median LOS (discharge)	2.0 (1.9 - 2.2)	3.9 (3.6 - 4.1)	6.2 (5.8 - 6.7)	6.6 (6.3 - 7.1)
**May-21**				
HFR	1.3% (0.7 - 2.4%)	11.5% (8.7 - 15.4%)	23.8% (16.8 - 33.7%)	35.1% (25.9 - 47.5%)
Median LOS (death)	10.0 (6.9 - 11.9)	16.3 (15.3 - 18.9)	5.3 (4.1 - 5.8)	9.2 (7.4 - 10.7)
Median LOS (discharge)	2.2 (2.0 - 2.4)	3.4 (3.1 - 3.7)	6.8 (5.7 - 8.1)	7.3 (6.8 - 7.9)
**Jun-21**				
HFR	1.8% (1.4 - 2.5%)	11.1% (9.3 - 13.3%)	28.1% (23.3 - 34.0%)	28.4% (22.0 - 36.7%)
Median LOS (death)	13.5 (11.4 - 15.7)	10.6 (10.3 - 11.1)	10.2 (9.7 - 11.0)	7.7 (7.0 - 9.1)
Median LOS (discharge)	2.0 (1.9 - 2.1)	3.8 (3.6 - 4.0)	7.6 (6.9 - 8.3)	8.5 (7.2 - 9.7)
**Jul-21**				
HFR	2.2% (1.9 - 2.5%)	11.0% (10.1 - 12.0%)	25.9% (23.4 - 28.5%)	30.6% (27.0 - 34.7%)
Median LOS (death)	11.7 (11.4 - 12.2)	10.2 (9.9 - 10.5)	8.1 (7.6 - 8.7)	8.9 (8.5 - 9.5)
Median LOS (discharge)	2.1 (2.1 - 2.2)	4.3 (4.2 - 4.4)	6.5 (6.2 - 6.8)	6.5 (6.2 - 6.9)
**Aug-21**				
HFR	2.7% (2.4 - 3.1%)	12.1% (11.2 - 13.0%)	26.4% (24.4 - 28.6%)	29.4% (26.4 - 32.8%)
Median LOS (death)	12.7 (12.3 - 13.1)	9.4 (9.1 - 9.7)	8.1 (7.8 - 8.5)	9.1 (8.5 - 9.7)
Median LOS (discharge)	2.2 (2.1 - 2.2)	4.2 (4.1 - 4.3)	6.4 (6.2 - 6.7)	7.0 (6.7 - 7.3)
**Sep-21**				
HFR	2.4% (2.0 - 2.8%)	10.5% (9.6 - 11.5%)	24.6% (22.6 - 26.9%)	26.8% (23.8 - 30.1%)
Median LOS (death)	8.3 (8.1 - 8.7)	7.8 (7.6 - 8.1)	7.0 (6.8 - 7.3)	7.5 (7.1 - 7.9)
Median LOS (discharge)	2.2 (2.2 - 2.3)	4.4 (4.3 - 4.5)	6.7 (6.5 - 6.9)	6.8 (6.6 - 7.0)

Supplementary table 8: Hospitalised fatality risk and median length of stay (days) in hospital prior to death or discharge by month of hospital admission and measure of hospital load (unadjusted for other covariates). Estimates are replaced with a dash (-) where insufficient information was available. Figures in brackets represent 95% confidence intervals.

**Month of hospital admission**	**(0,20]**	**(20,40]**	**(40,60]**
**Mar-20**			
HFR	38.8% (36.6 - 41.0%)	40.1% (38.5 - 41.7%)	40.0% (38.3 - 41.9%)
Median LOS (death)	6.1 (5.9 - 6.2)	5.7 (5.6 - 5.9)	5.2 (5.1 - 5.4)
Median LOS (discharge)	5.6 (5.5 - 5.8)	6.0 (5.9 - 6.1)	6.0 (5.9 - 6.2)
**Apr-20**			
HFR	34.3% (32.6 - 36.0%)	37.3% (36.2 - 38.4%)	37.8% (36.6 - 39.0%)
Median LOS (death)	5.6 (5.5 - 5.8)	5.3 (5.2 - 5.4)	5.3 (5.2 - 5.4)
Median LOS (discharge)	6.0 (5.8 - 6.1)	5.9 (5.8 - 5.9)	5.7 (5.6 - 5.8)
**May-20**			
HFR	28.4% (27.1 - 29.8%)	33.6% (31.5 - 35.7%)	27.9% (23.4 - 33.3%)
Median LOS (death)	6.5 (6.4 - 6.7)	6.6 (6.4 - 6.9)	6.4 (6.0 - 7.0)
Median LOS (discharge)	5.3 (5.1 - 5.4)	5.9 (5.7 - 6.1)	6.2 (5.6 - 7.0)
**Jun-20**			
HFR	20.3% (18.7 - 22.0%)	28.4% (21.5 - 37.4%)	11.9% (5.2 - 27.1%)
Median LOS (death)	7.8 (7.5 - 8.1)	6.8 (6.4 - 7.7)	15.0 (7.2 - 16.9)
Median LOS (discharge)	4.8 (4.6 - 4.9)	6.3 (5.6 - 7.1)	6.7 (5.4 - 7.9)
**Jul-20**			
HFR	13.2% (11.3 - 15.5%)	-	-
Median LOS (death)	10.3 (9.6 - 10.9)	-	-
Median LOS (discharge)	4.0 (3.8 - 4.2)	-	-
**Aug-20**			
HFR	12.4% (10.3 - 14.9%)	14.4% (4.0 - 52.0%)	-
Median LOS (death)	9.9 (9.2 - 10.9)	5.0 (2.5 - 6.4)	-
Median LOS (discharge)	3.1 (2.9 - 3.3)	4.5 (2.0 - 5.5)	-
**Sep-20**			
HFR	13.7% (12.5 - 15.1%)	19.6% (17.4 - 22.2%)	17.6% (11.0 - 28.3%)
Median LOS (death)	9.2 (8.9 - 9.6)	8.9 (8.5 - 9.4)	4.0 (2.2 - 4.9)
Median LOS (discharge)	3.9 (3.8 - 4.0)	4.7 (4.5 - 4.9)	2.8 (2.4 - 3.6)
**Oct-20**			
HFR	15.9% (14.9 - 17.1%)	19.3% (18.1 - 20.6%)	20.6% (19.4 - 21.9%)
Median LOS (death)	9.1 (8.8 - 9.4)	8.7 (8.4 - 9.0)	8.4 (8.1 - 8.7)
Median LOS (discharge)	4.4 (4.3 - 4.4)	4.3 (4.2 - 4.4)	4.5 (4.4 - 4.6)
**Nov-20**			
HFR	17.4% (16.4 - 18.6%)	20.8% (19.7 - 21.9%)	20.8% (19.8 - 21.8%)
Median LOS (death)	9.2 (8.9 - 9.5)	8.7 (8.5 - 9.0)	8.3 (8.1 - 8.5)
Median LOS (discharge)	4.9 (4.8 - 5.0)	4.9 (4.8 - 5.0)	4.7 (4.6 - 4.8)
**Dec-20**			
HFR	19.6% (17.9 - 21.5%)	22.7% (21.8 - 23.5%)	23.7% (22.8 - 24.6%)
Median LOS (death)	9.5 (9.1 - 9.9)	8.7 (8.6 - 8.9)	8.4 (8.2 - 8.6)
Median LOS (discharge)	4.9 (4.7 - 5.0)	5.0 (4.9 - 5.0)	5.2 (5.1 - 5.3)
**Jan-21**			
HFR	-	19.4% (17.2 - 21.8%)	22.2% (21.3 - 23.1%)
Median LOS (death)	-	8.1 (7.6 - 8.6)	8.1 (7.9 - 8.2)
Median LOS (discharge)	-	4.8 (4.7 - 5.0)	4.6 (4.5 - 4.6)

Supplementary table 8 continued from previous page

**Feb-21**			
HFR	14.1% (12.9 - 15.3%)	17.5% (16.7 - 18.3%)	20.0% (19.0 - 21.1%)
Median LOS (death)	7.9 (7.7 - 8.2)	8.0 (7.9 - 8.2)	7.3 (7.1 - 7.6)
Median LOS (discharge)	4.3 (4.2 - 4.4)	4.5 (4.5 - 4.6)	4.6 (4.5 - 4.7)
**Mar-21**			
HFR	10.1% (9.2 - 11.0%)	12.2% (10.5 - 14.2%)	12.4% (5.5 - 28.3%)
Median LOS (death)	8.8 (8.5 - 9.2)	8.3 (7.8 - 9.1)	14.5 (15.9 - 18.1)
Median LOS (discharge)	3.6 (3.5 - 3.6)	3.9 (3.7 - 4.0)	7.0 (5.4 - 9.6)
**Apr-21**			
HFR	8.9% (7.6 - 10.3%)	-	-
Median LOS (death)	8.6 (8.2 - 9.0)	-	-
Median LOS (discharge)	3.2 (3.1 - 3.3)	-	-
**May-21**			
HFR	8.9% (7.4 - 10.7%)	5.6% (2.4 - 13.0%)	-
Median LOS (death)	10.1 (9.6 - 10.8)	11.3 (5.5 - 17.5)	-
Median LOS (discharge)	2.8 (2.6 - 2.9)	3.3 (2.7 - 3.8)	-
**Jun-21**			
HFR	8.0% (7.0 - 9.1%)	9.0% (7.1 - 11.3%)	4.7% (1.8 - 12.3%)
Median LOS (death)	10.6 (10.2 - 11.0)	10.0 (9.5 - 10.7)	8.0 (4.5 - 11.3)
Median LOS (discharge)	2.7 (2.7 - 2.8)	2.5 (2.3 - 2.6)	4.2 (3.2 - 4.9)
**Jul-21**			
HFR	8.2% (7.5 - 8.9%)	10.0% (9.2 - 10.9%)	8.2% (7.1 - 9.4%)
Median LOS (death)	10.2 (9.9 - 10.5)	10.1 (9.8 - 10.4)	8.5 (7.9 - 9.1)
Median LOS (discharge)	3.0 (2.9 - 3.0)	3.1 (3.1 - 3.2)	3.0 (2.9 - 3.1)
**Aug-21**			
HFR	9.8% (9.0 - 10.5%)	11.3% (10.5 - 12.1%)	11.8% (10.4 - 13.4%)
Median LOS (death)	9.9 (9.5 - 10.2)	9.7 (9.4 - 9.9)	8.9 (8.5 - 9.4)
Median LOS (discharge)	3.4 (3.4 - 3.5)	3.2 (3.1 - 3.3)	2.8 (2.7 - 2.9)
**Sep-21**			
HFR	9.8% (9.0 - 10.6%)	11.2% (10.3 - 12.1%)	11.9% (10.1 - 14.1%)
Median LOS (death)	7.6 (7.4 - 7.9)	7.5 (7.2 - 7.7)	8.2 (7.9 - 8.5)
Median LOS (discharge)	3.5 (3.4 - 3.6)	3.8 (3.7 - 3.8)	3.2 (3.1 - 3.4)

Supplementary table 8 continued

**Month of hospital admission**	**(60,80]**	**(80,90]**	**(90,100]**
**Mar-20**			
HFR	41.9% (39.7 - 44.2%)	41.9% (37.9 - 46.3%)	44.9% (39.6 - 50.9%)
Median LOS (death)	5.5 (5.3 - 5.6)	5.5 (5.2 - 5.9)	4.2 (3.8 - 4.7)
Median LOS (discharge)	5.8 (5.6 - 6.0)	6.5 (6.2 - 6.9)	5.0 (4.6 - 5.4)
**Apr-20**			
HFR	38.4% (37.0 - 39.8%)	38.4% (35.7 - 41.4%)	38.6% (35.7 - 41.7%)
Median LOS (death)	4.9 (4.8 - 5.0)	4.7 (4.4 - 4.9)	5.2 (4.9 - 5.4)
Median LOS (discharge)	5.7 (5.6 - 5.8)	5.7 (5.5 - 5.9)	6.9 (6.7 - 7.2)
**May-20**			
HFR	16.2% (8.9 - 29.4%)	9.1% (1.4 - 58.9%)	4.3% (0.6 - 29.6%)
Median LOS (death)	8.3 (4.1 - 10.9)	21.0 (21.0 - 21.0)	2.0 (2.0 - 2.0)
Median LOS (discharge)	8.5 (7.6 - 11.0)	10.0 (6.2 - 11.4)	11.3 (7.2 - 12.6)
**Jun-20**			

Supplementary table 8 continued from previous page

HFR	8.4% (2.8 - 24.8%)	-	-
Median LOS (death)	9.3 (6.1 - 11.8)	-	-
Median LOS (discharge)	6.3 (4.6 - 7.8)	-	-
**Jul-20**			
HFR	-	-	-
Median LOS (death)	-	-	-
Median LOS (discharge)	-	-	-
**Aug-20**			
HFR	-	-	-
Median LOS (death)	-	-	-
Median LOS (discharge)	-	-	-
**Sep-20**			
HFR	11.1% (1.8 - 70.5%)	-	-
Median LOS (death)	6.0 (6.0 - 6.0)	-	-
Median LOS (discharge)	2.0 (0.0 - 2.6)	-	-
**Oct-20**			
HFR	20.9% (19.2 - 22.8%)	23.1% (19.9 - 26.9%)	20.0% (15.5 - 25.9%)
Median LOS (death)	8.0 (7.7 - 8.4)	6.8 (6.1 - 7.6)	7.3 (5.6 - 8.7)
Median LOS (discharge)	4.8 (4.6 - 4.9)	5.2 (5.0 - 5.5)	5.9 (5.6 - 6.3)
**Nov-20**			
HFR	22.8% (21.5 - 24.2%)	23.6% (21.5 - 25.8%)	23.6% (21.1 - 26.4%)
Median LOS (death)	7.9 (7.6 - 8.2)	7.4 (7.0 - 7.9)	8.0 (7.6 - 8.5)
Median LOS (discharge)	4.8 (4.7 - 4.9)	5.1 (4.9 - 5.2)	5.2 (5.0 - 5.4)
**Dec-20**			
HFR	22.2% (21.2 - 23.3%)	22.7% (21.2 - 24.3%)	19.8% (17.9 - 21.8%)
Median LOS (death)	8.2 (8.0 - 8.4)	8.0 (7.8 - 8.4)	7.9 (7.5 - 8.3)
Median LOS (discharge)	5.3 (5.2 - 5.4)	4.7 (4.6 - 4.8)	4.7 (4.6 - 4.9)
**Jan-21**			
HFR	22.1% (21.5 - 22.7%)	23.2% (22.5 - 24.0%)	23.8% (23.2 - 24.4%)
Median LOS (death)	7.5 (7.4 - 7.6)	7.2 (7.1 - 7.4)	7.5 (7.4 - 7.6)
Median LOS (discharge)	5.0 (4.9 - 5.0)	5.0 (4.9 - 5.0)	4.9 (4.9 - 5.0)
**Feb-21**			
HFR	19.1% (16.9 - 21.7%)	19.6% (10.1 - 38.0%)	11.8% (3.2 - 43.2%)
Median LOS (death)	7.7 (7.3 - 8.1)	12.0 (5.4 - 14.5)	20.0 (7.7 - 26.8)
Median LOS (discharge)	5.8 (5.5 - 6.0)	8.0 (7.5 - 8.8)	17.0 (6.5 - 20.0)
**Mar-21**			
HFR	4.8% (0.7 - 32.2%)	-	-
Median LOS (death)	2.0 (2.0 - 2.0)	-	-
Median LOS (discharge)	3.0 (1.5 - 4.7)	-	-
**Apr-21**			
HFR	-	-	-
Median LOS (death)	-	-	-
Median LOS (discharge)	-	-	-
**May-21**			
HFR	-	-	-
Median LOS (death)	-	-	-
Median LOS (discharge)	-	-	-
**Jun-21**			
HFR	-	-	-
Median LOS (death)	-	-	-

Supplementary table 8 continued from previous page

Median LOS (discharge)	-	-	-
**Jul-21**			
HFR	5.9% (4.1 - 8.5%)	-	-
Median LOS (death)	10.4 (8.5 - 11.8)	-	-
Median LOS (discharge)	2.8 (2.6 - 3.0)	-	-
**Aug-21**			
HFR	10.8% (6.8 - 17.1%)	18.3% (9.8 - 34.2%)	3.9% (0.6 - 26.4%)
Median LOS (death)	6.7 (4.4 - 8.3)	4.0 (3.8 - 5.1)	5.0 (5.0 - 5.0)
Median LOS (discharge)	3.2 (2.7 - 3.6)	1.9 (1.6 - 2.2)	1.2 (0.8 - 1.4)
**Sep-21**			
HFR	11.7% (7.6 - 18.0%)	-	-
Median LOS (death)	7.0 (4.1 - 11.9)	-	-
Median LOS (discharge)	3.2 (2.6 - 3.9)	-	-

Supplementary table 9: Hospitalised fatality sub-distribution hazard ratio by month of hospital admission, stratified by age group, region of residence and vaccination status, with regression adjustment (main effects) on sex, ethnicity, IMD quintile, CCI and hospital load. Figures in brackets represent 95% confidence intervals.

**Month of hospital admission**	**Hazard ratio**
Mar-20	2.30 (2.08, 2.55)
Apr-20	1.93 (1.75, 2.13)
May-20	1.33 (1.20, 1.48)
Jun-20	1 (reference category)
Jul-20	0.74 (0.61, 0.91)
Aug-20	0.87 (0.69, 1.08)
Sep-20	1.07 (0.94, 1.21)
Oct-20	1.11 (1.00, 1.23)
Nov-20	1.14 (1.03, 1.26)
Dec-20	1.30 (1.17, 1.44)
Jan-21	1.32 (1.19, 1.46)
Feb-21	1.19 (1.07, 1.32)
Mar-21	1.00 (0.88, 1.14)
Apr-21	0.90 (0.75, 1.10)
May-21	1.20 (0.97, 1.50)
Jun-21	1.43 (1.23, 1.68)
Jul-21	1.31 (1.16, 1.47)
Aug-21	1.31 (1.17, 1.48)
Sep-21	1.15 (1.02, 1.30)

Supplementary table 10: Hospitalised fatality sub-distribution hazard ratios by person characteristic, stratified by age group, PHE centre of residence and month of hospitalisation, with regression adjustment (main effects) on vaccination status, sex, ethnicity, IMD quintile, CCI and hospital load. January to September 2021. Figures in brackets represent 95% confidence intervals.

**Characteristic**	**Hazard ratio**
**Vaccination status**	
Unvaccinated	1 (reference category)
$<$21 days after first vaccination dose	0.93 (0.89 - 0.98)
$\geq$21 days after first vaccination dose	0.71 (0.67 - 0.77)
$\geq$14 days after second vaccination dose	0.56 (0.52 - 0.61)
**Sex**	
Male	1.28 (1.24 - 1.32)
Female	1 (reference category)
**Ethnicity**	
White	1 (reference category)
Asian	1.19 (1.13 - 1.25)
Black	0.90 (0.84 - 0.97)
Mixed/Other/Unknown	1.02 (0.94 - 1.10)
**Index of multiple deprivation**	
1st quintile (most deprived)	1.10 (1.05 - 1.15)
2nd quintile	1.09 (1.04 - 1.14)
3rd quintile	1.06 (1.01 - 1.11)
4th quintile	1.03 (0.98 - 1.09)
5th quintile (least deprived)	1 (reference category)
**Charleson comorbidity index**	
0	1 (reference category)
1-2	1.92 (1.83 - 2.01)
3-4	2.79 (2.65 - 2.94)
5+	3.46 (3.27 - 3.67)
**Hospital load at time of admission (as proportion of busiest week)**	
0-20%	1 (reference category)
20-40%	1.11 (1.04 - 1.19)
40-60%	1.18 (1.10 - 1.28)
60-80%	1.15 (1.05 - 1.25)
80-95%	1.22 (1.11 - 1.33)
95-100%	1.23 (1.12 - 1.34)

References

1. Andersen PK, Geskus RB, de Witte T, Putter H. Competing risks in epidemiology: possibilities and pitfalls. Int J Epidemiol. 2012;41: 861–870.

2. Aalen OO, Johansen S. An Empirical Transition Matrix for Non-Homogeneous Markov Chains Based on Censored Observations. Scand Stat Theory Appl. 1978;5: 141–150.

3. Fine JP, Gray RJ. A proportional hazards model for the subdistribution of a competing risk. J Am Stat Assoc. 1999.

4. Bradburn MJ, Clark TG, Love SB, Altman DG. Survival analysis part II: multivariate data analysis–an introduction to concepts and methods. Br J Cancer. 2003;89: 431–436.

5. Seaman SR, Nyberg T, Overton CE, Pascall D, Presanis AM, De Angelis D. Adjusting for time of infection or positive test when estimating the risk of a post-infection outcome in an epidemic. 2021. doi:10.1101/2021.08.13.21262014
