# Supplementary material for: Trends in COVID-19 hospital outcomes in England before and after vaccine introduction, a cohort study
Source: Nat Commun. 2022 Aug 17;13:4834. doi: 10.1038/s41467-022-32458-y (PMC9382625; doi:10.1038/s41467-022-32458-y)
Supplement: Supplementary file 3 — Reporting Summary [file 41467_2022_32458_MOESM3_ESM.pdf]

nature portfolio

Corresponding author(s): Peter Kirwan

Last updated by author(s): Jul 18, 2022

Reporting Summary

Nature Portfolio wishes to improve the reproducibility of the work that we publish. This form provides structure for consistency and transparency in reporting. For further information on Nature Portfolio policies, see our Editorial Policies and the Editorial Policy Checklist.

Statistics

For all statistical analyses, confirm that the following items are present in the figure legend, table legend, main text, or Methods section.

n/a	Confirmed	
☐	☒	The exact sample size (*n*) for each experimental group/condition, given as a discrete number and unit of measurement
☐	☒	A statement on whether measurements were taken from distinct samples or whether the same sample was measured repeatedly
☐	☒	The statistical test(s) used AND whether they are one- or two-sided<br>*Only common tests should be described solely by name; describe more complex techniques in the Methods section.*
☐	☒	A description of all covariates tested
☐	☒	A description of any assumptions or corrections, such as tests of normality and adjustment for multiple comparisons
☐	☒	A full description of the statistical parameters including central tendency (e.g. means) or other basic estimates (e.g. regression coefficient) AND variation (e.g. standard deviation) or associated estimates of uncertainty (e.g. confidence intervals)
☒	☐	For null hypothesis testing, the test statistic (e.g. *F*, *t*, *r*) with confidence intervals, effect sizes, degrees of freedom and *P* value noted<br>*Give P values as exact values whenever suitable.*
☒	☐	For Bayesian analysis, information on the choice of priors and Markov chain Monte Carlo settings
☒	☐	For hierarchical and complex designs, identification of the appropriate level for tests and full reporting of outcomes
☒	☐	Estimates of effect sizes (e.g. Cohen's *d*, Pearson's *r*), indicating how they were calculated

Our web collection on statistics for biologists contains articles on many of the points above.

Software and code

Policy information about availability of computer code

Data collection	No software was used for data collection. Data files (typically XML format) were reported to secure data warehouses that store patient-level information in line with national standards using existing UKHSA and NHS Digital clinical reporting mechanisms. Guidance to data providers (NHS trusts) is provided by NHS Digital (https://digital.nhs.uk).
Data analysis	Statistical models were implemented using R version 4.1.1 (R Foundation for Statistical Computing, Vienna, Austria) and the open source packages survival 3.2-12, and matrixStats 0.60.0.<br>Code for the survival and matrixStats R packages is available from the Comprehensive R Archive Network (https://cran.r-project.org/).<br>R code written to process the data, implement the statistical analysis, and produce the figures and tables is available from: https://github.com/pkirwan/COVID-hospital-outcomes.

For manuscripts utilizing custom algorithms or software that are central to the research but not yet described in published literature, software must be made available to editors and reviewers. We strongly encourage code deposition in a community repository (e.g. GitHub). See the Nature Portfolio guidelines for submitting code & software for further information.

Data

Policy information about availability of data

All manuscripts must include a data availability statement. This statement should provide the following information, where applicable:

- Accession codes, unique identifiers, or web links for publicly available datasets
- A description of any restrictions on data availability
- For clinical datasets or third party data, please ensure that the statement adheres to our policy

The data used in this study are protected data. These data are not publicly available because the information is personal or special category personal data, and there is risk of 're-identification' of data that has been anonymised by data matching, inference or deductive disclosure.
Access to protected data is subject to robust governance protocols, where it is lawful, ethical and safe to do so. Individuals and organisations wishing to request access to data used in this study, from the NHS England Secondary Uses Service (SUS), Emergency Care Dataset for England (ECDS), UKHSA deaths dataset, UKHSA National Immunisation Management Service (NIMS), or UKHSA Second Generation Surveillance System (SGSS) can make a request directly to NHS Digital (https://digital.nhs.uk/services/data-access-request-service-dars) or to UKHSA (https://www.gov.uk/government/publications/accessing-ukhsa-protected-data). Access to protected data is always strictly controlled using legally binding data sharing contracts.
Requests for underlying data cannot be granted by the authors because the data were acquired under licence/data sharing agreement from NHS Digital and UKHSA, for which conditions of use (and further use) apply.

Human research participants

Policy information about studies involving human research participants and Sex and Gender in Research.

Reporting on sex and gender	Data on sex were obtained from the Secondary Uses Services dataset (https://digital.nhs.uk/services/secondary-uses-service-sus) which records the sex of individuals registered at birth, as defined by the NHS data model and dictionary. The term sex is used throughout the study to refer to this covariate. Findings are described by sex where appropriate, and frequencies in each group are presented in Table 1.
Population characteristics	Table 1 in the manuscript presents patient characteristics for the study population by age, sex, ethnicity, region, deprivation, vaccination status and comorbidity burden. The population characteristics are described within the Results section.
Recruitment	Data comprised all new hospital admissions for COVID-19 reported in England, with the only exclusion criteria being hospital-onset COVID. Numbers of reported admissions were compared with the NHS weekly COVID-19 admissions data to ensure data were representative. No evidence of bias due to under-reporting was seen. We found no systematic bias due to misreporting of person characteristics. Linked information was used to minimise missing data bias, counts of records with missing information are included
Ethics oversight	This study does not contain patient identifiable data. Consent from individuals involved in this study was not required. The mandatory surveillance systems used in this study, NHS England Secondary Uses Service (SUS), Emergency Care Dataset for England (ECDS), UKHSA deaths dataset, UKHSA National Immunisation Management Service (NIMS), and UKHSA Second Generation Surveillance System (SGSS), are approved by the Department of Health and Social Care. Data were collected with permissions granted under Regulation 3 of The Health Service (Control of Patient Information) Regulations 2002, and without explicit patient permission under Section 251 of the NHS Act 2006.

Note that full information on the approval of the study protocol must also be provided in the manuscript.

Field-specific reporting

Please select the one below that is the best fit for your research. If you are not sure, read the appropriate sections before making your selection.

☒ Life sciences ☐ Behavioural & social sciences ☐ Ecological, evolutionary & environmental sciences

For a reference copy of the document with all sections, see nature.com/documents/nr-reporting-summary-flat.pdf

Life sciences study design

All studies must disclose on these points even when the disclosure is negative.

Sample size	Data comprised all new hospital admissions for COVID-19 in England between March 2020 and September 2021 (n=259,727). These data were a comprehensive cohort of all individuals admitted to hospital, no sample size calculations were performed since no sampling of the data was undertaken. Confidence intervals which reflect the uncertainty in our estimates are included.
Data exclusions	Hospital-onset COVID-19 (i.e. infection occurring in hospital) cases were excluded from the main study: those with hospital-onset infection (n=194,888) tended to be older and have longer lengths of stay than the community-onset cases considered in this study. A comparison of the study population to hospital-onset cases is included in the manuscript (Table 1).<br>Patient records with inconsistent date information (n=2) or missing demographic information (n=302) were excluded.

Replication	Findings were replicated on two separate machines with independent checking of the associated R commands and data processing procedures. This replication was successful with very similar or identical estimates obtained.<br>Efforts to reproduce findings through the use of different datasets were not pursued since the data were a comprehensive cohort of all hospital admissions and estimates were generated by considering all admissions.
Randomization	This was an observational cohort study of all hospital admissions for COVID-19 in England. Randomisation was not relevant to this study as no allocation to experimental groups was undertaken.
Blinding	This was an observational cohort study of all hospital admissions for COVID-19 in England. Blinding was not relevant to this study as no allocation to experimental groups was undertaken.

Reporting for specific materials, systems and methods

We require information from authors about some types of materials, experimental systems and methods used in many studies. Here, indicate whether each material, system or method listed is relevant to your study. If you are not sure if a list item applies to your research, read the appropriate section before selecting a response.

Materials & experimental systems

n/a	Involved in the study	
☒	☐	Antibodies
☒	☐	Eukaryotic cell lines
☒	☐	Palaeontology and archaeology
☒	☐	Animals and other organisms
☒	☐	Clinical data
☒	☐	Dual use research of concern

Methods

n/a	Involved in the study	
☒	☐	ChIP-seq
☒	☐	Flow cytometry
☒	☐	MRI-based neuroimaging
